# The insulator EACBE regulates V(D)J recombination of Tcrd gene by modulating chromatin organization

**DOI:** 10.3389/fimmu.2025.1613621

**Published:** 2025-07-17

**Authors:** Yongchang Zhu, Ranran Dai, Hao Zhao, Junwei Luo, Keyi Li, Wei Xue, Litao Qin, Hongyuan Pan, Shixiu Liao, Bingtao Hao

**Affiliations:** ^1^ Henan Key Provincial Laboratory of Genetic Diseases and Functional Genomics, People’s Hospital of Zhengzhou University, Zhengzhou University, Zhengzhou, China; ^2^ Department of Immunology, School of Basic Medical Sciences, Zhengzhou University, Zhengzhou, China; ^3^ RNA Biomedical Institute, Sun Yat-Sen Memorial Hospital, Zhongshan School of Medicine, Sun Yat-Sen University, Guangzhou, China; ^4^ Center for Stem Cell Biology and Tissue Engineering, Key Laboratory for Stem Cells and Tissue Engineering, Ministry of Education, Zhongshan School of Medicine, Sun Yat-Sen University, Guangzhou, China; ^5^ Cancer Research Institute, School of Basic Medical Sciences, Southern Medical University, Guangzhou, China; ^6^ Henan Eye Institute, Henan Academy of Innovations in Medical Science, Zhengzhou, China

**Keywords:** CTCF binding element, T cell receptor, V(D)J recombination, chromatin architecture, chromatin activity

## Abstract

T cell receptor (TCR) diversity, essential for the recognition of a wide array of antigens, is generated through V(D)J recombination. The *Tcra* and *Tcrd* genes reside within a shared genomic locus, with *Tcrd* rearrangement occurring first in the double-negative (DN) stage during thymocyte development. Elucidating the regulatory mechanisms governing *Tcrd* rearrangement is therefore crucial for understanding the developmental coordination of both *Tcrd* and *Tcra* rearrangements. Chromatin architecture, orchestrated by CTCF-cohesin complexes and their binding sites, plays a fundamental role in regulating V(D)J recombination of antigen receptor genes. In this study, we report that EACBE, a CTCF binding element (CBE) located downstream of the *Tcra*-*Tcrd* locus, regulates *Tcrd* rearrangement. EACBE promotes the usage of proximal V_δ_ gene segments by facilitating spatial proximity between the *Tcrd* recombination centre and these V_δ_ elements. Notably, EACBE counteracts the insulating effects of INTs, two CBEs that demarcate the proximal V region from the D_δ_-J_δ_-C_δ_ cluster, thereby enabling effective chromatin extrusion. Furthermore, EACBE indirectly shapes the *Tcra* repertoire through its influence on *Tcrd* rearrangement. These findings reveal a novel regulatory axis involving special chromatin configuration and highlight distinct roles for specific CTCF binding sites in modulating antigen receptor gene assembly.

## 1 Introduction

The adaptive immune system relies on T and B lymphocytes to detect and respond to foreign pathogens through highly diverse surface antigen receptors (1). T cell development in the thymus progresses through three stages: double negative (DN), double positive (DP), and single positive (SP). T cell receptor (TCR) diversity arises from recombination of variable (V), diversity (D), and joining (J) gene segments, flanked by recombination signal sequences (RSSs) recognized by the RAG1/2 recombinase complex. Among the four genes (*Tcra*, *Tcrb*, *Tcrg*, and *Tcrd*), *Tcra* and *Tcrd* are uniquely co-located within a single genomic locus on chromosome 14 in mice. The *Tcrd* gene lies between the V_α_ and J_α_ gene clusters and shares a subset of V gene segments with *Tcrα* (2). Chromatin accessibility has been shown to regulate the recombination initiation, with germline transcription increasing accessibility of RSSs for RAG binding (3). This establishes a mechanistic link between transcriptional regulation and V(D)J recombination. Enhancers E_δ_ and E_α_ play essential role in promoting transcription and recombination of *Tcrd* and *Tcra*, respectively (4–6).

Chromatin is organized in a highly structured and hierarchical manner within the nucleus, and this organization is tightly regulated by architectural proteins such as CTCF and cohesion (7). CTCF, a conserved 11 zinc finger protein, binds to CBEs and mediates a range of gene regulatory functions, including transcriptional insulation and long-distance chromatin interactions (8–10). Cohesin, a ring-like tetrameric complex, is best known for its role in sister chromatid cohesion during mitosis but also contributes significantly to higher-order chromatin organization and facilitates genome-wide chromatin interactions (11, 12). CTCF and cohesin frequently colocalize at genomic sites, where convergent CBEs serves as anchors for the formation of chromatin loops (13–16). Targeted degradation or genetic ablation of CTCF or cohesin disrupts these interactions, underscoring their essential roles in genome topology (17, 18). In the context of antigen receptor gene rearrangement, the juxtaposition of V gene segments with (D)J segments is a prerequisite for effective V(D)J recombination. Chromatin immunoprecipitation (ChIP) analyses have demonstrated that CTCF and cohesin colocalize at V segments and cis-regulatory elements in the *Tcra*-*Tcrd* locus (19). Notably, deletion of either CTCF or cohesin in DP thymocytes impaired *Tcra* rearrangement by disrupting chromatin loops between regulatory elements (20, 21).

CBEs and their coordinated interactions play a crucial role in regulating the spatial organization and rearrangement of antigen receptor genes. Specifically, IGCR1 and 3’CBE, which consist of two CBEs and a tandem array of ten CBEs, respectively, are located within the immunoglobulin heavy chain (*Igh*) locus. The chromatin loop structure formed by the interaction between IGCR1 and 3’CBE restricts the spatial proximity between the 3’V_H_ region and the DJ_H_ region, thereby orchestrating *Igh* rearrangement (22–25). In the *Tcra*-*Tcrd* locus, two CBEs, INT1 and INT2, collectively known as INTs, are positioned between the proximal V_α_ region and the first V_δ_ gene, *Trdv4*. At the DN stage, INTs interacts with the CBE in the TEA promoter to form a chromatin loop that encompasses the *Tcrd* recombination center, thereby regulating *Tcrd* rearrangement. Deletion of INTs results in a significant increase in the usage of *Trdv2-2*, while the usage of distal V_δ_ segments is reduced. This shift reflects enhanced proximity between *Trdv2–2* and the *Tcrd* recombination center. These findings indicate that the loop formed by INTs and TEA CBE restricts the rearrangement of proximal V_δ_ segments, thus increasing *Tcrd* diversity (26).

During thymocyte development, *Tcrd* undergoes rearrangement at the DN stage, while *Tcra* rearranges at the DP stage, with *Tcrd* rearrangement proceding *Tcra*. The V_δ_ to DJ_δ_ rearrangement results in the deletion of the genomic region between the used V_δ_ segment and the D_δ_-J_δ_-C_δ_ region, which subsequently affects *Tcra* rearrangement. Previous studies have shown that *Tcra* rearrangement initiates from the proximal V_α_ and J_α_ genes, progressively extending towards the distal regions. Consequently, *Tcrd* rearrangement, especially the rearrangements of V_δ_ segments in the repetitive V region, promotes the usage of V_α_ segments, thereby increasing the diversity of the TCRα repertoire. Deletion of the INTs has been shown to impair *Tcra* rearrangement, likely due to defects in *Tcrd* rearrangement (26). Through the ablation of *Tcrd* recombination, Danielle J et al. discovered that *Tcrd* rearrangement enhanced the diversity of the primary V_α_ rearrangement in mice (27). Therefore, *Tcrd* rearrangement plays a crucial role in maintaining the diversity of the *Tcra* repertoire.

Two CBEs have been identified just downstream of the enhancer E_α_, referred to as EACBE. Our previous findings have shown that EACBE regulates *Tcra* rearrangement (28). Previous studies have shown that E_α_ is primed but inactive during the DN stage, and no evidence suggests that E_α_ contributes *Tcrd* rearrangement (6, 29). However, our prior research indicated that EACBE deletion also affects the *Tcrd* repertoire. Specifically, in EACBE^-/-^ thymocytes, the usage of proximal V_δ_ segments, such as *Trdv2–2* and *Trdv1*, was reduced, whereas the usage of *Trdv5* and distal V_δ_ genes was increased (28), contrasting with the effects of INTs on *Tcrd* rearrangement. Nonetheless, it has been documented that E_α_ influences the expression of rearranged *Tcrd* (6, 30). The question of whether the impact of EACBE on the *Tcrd* repertoire is due to direct effects on rearrangement or post-rearrangement expression requires further investigation, as the underlying mechanisms remain unclear. Additionally, it remains to be determined whether the influence of EACBE on *Tcra* rearrangement at the DP stage is a consequence of its effect on *Tcrd* rearrangement at the DN stage.

To address these issues, we conducted this study on DN cells derived from EACBE knockout mice. Our findings indicate that EACBE has a direct impact on *Tcrd* rearrangement. Additionally, EACBE indirectly influences the V_α_ usage in DP cells by modulating *Tcrd* rearrangement, specifically affecting the diversity of Trav14-related TCR. ATAC-seq and germline transcription results demonstrate that EACBE deletion slightly reduces the chromatin activity of *Trdv2-2*. Furthermore, we provide evidence that EACBE facilitates the *Tcrd* recombination center to overcome the isolation imposed by INTs, thereby enhancing its interaction with the proximal V_δ_ region.

## 2 Materials and methods

### 2.1 Mice

Mice used for all experiments were 4 to 8-week-old of mixed sex and housed in a specific-pathogen-free facility managed by the Southern Medical University Division of Laboratory Animal Center. EACBE^-/-^, *Rag1* ^-/-^, *Rag2* ^-/-^, EACBE^-/-^ *Rag2* ^-/-^and EACBE^-/-^ *Rag1* ^-/-^ mice had been previously characterized (PMID: 32853367, 37534534). All procedures involving mice were conducted in strict compliance with the protocols sanctioned by the Institutional Animal Care and Use Committee at Southern Medical University.

### 2.2 Cell collection

Thymus glands were carefully harvested and homogenized in MACS buffer. Thymocytes were filtered through a 40 μm nylon mesh to obtain a single-cell suspension. For LAM-HTGTS analysis, DN thymocytes (Thy1.2^+^, CD4^-^, CD8^-^) and DP thymocytes (Thy1.2^+^, CD4^+^, CD8^+^) were sorted from WT or EACBE^-/-^ mice. Rag-deficient DN thymocytes are directly isolated from *Rag1* ^-/-^ and EACBE^-/-^ *Rag1* ^-/-^ mice.

### 2.3 Flow cytometry and cell sorting

Unless otherwise specified, all antibodies were procured from Biolegend. DN and DP cells were sorted through staining with antibodies targeting CD4 (RM4–5), CD8 (53–6.7), and Thy1.2 (53–2.1). The γδ-T cells in the thymus, spleen, and lymph nodes were identified using anti-γδ-T (GL3) and CD3 (145–2C11) antibodies. Data acquisition was performed using a BD FACSCanto II flow cytometer configured for eight-color analysis.

### 2.4 PCR and Southern blot analysis of Vδ usage

Total thymocytes were lysed by incubation in a buffer containing 10 mM Tris–HCl (pH 8.0), 150 mM NaCl, 10 mM EDTA, 0.4% (wt/vol) SDS, and 0.1 mg/ml proteinase K, maintained overnight at 37°C. Genomic DNA was subsequently isolated using phenol/chloroform extraction followed by ethanol precipitation. The polymerase chain reaction (PCR) was conducted under the following conditions: an initial denaturation at 95°C for 3 minutes; 30 cycles consisting of denaturation at 95°C for 30 seconds, annealing at 60°C for 30 seconds, and extension at 72°C for 1 minute; and a final extension at 72°C for 5 minutes. Following agarose gel electrophoresis and transfer to nylon membranes, PCR products were detected through hybridization with biotin-labeled oligonucleotide probes. The sequences of primers and probes are detailed in **Supplementary Table S1**.

### 2.5 3C-HTGTS

3C-HTGTS libraries were constructed using thymocytes isolated from *Rag1* ^-/-^ or EACBE^-/-^ *Rag1* ^-/-^ mice. For each experiment, three to four mice were utilized. The detailed methodology has been previously outlined (31). The sequences of the nested primer, and adapter-complementary primer are provided in **Supplementary Table S2**.

### 2.6 ATAC-seq

To analyze open chromatin regions, ATAC-seq was conducted utilizing DN thymocytes derived from *Rag1* ^-/-^ or EACBE^-/-^ *Rag1* ^-/-^ mice. Initially, approximately 5 × 10^4 cell pellets were washed once with cold PBS. Cells were lysed on ice for 3 minutes in 50 μl ice-cold Lysis Buffer, which comprised 10 mM Tris at pH 7.4, 10 mM NaCl, 3 mM MgCl2, 0.1% NP-40, 0.1% TWEEN 20, and 0.01% Digitonin dissolved in DEPC-treated water. Following lysis, the cells were resuspended in 1 ml of ice-cold RBS-Wash buffer containing 10 mM Tris at pH 7.4, 10 mM NaCl, 3 mM MgCl2, and 0.1% TWEEN 20, and then centrifuged at 4°C at 500 × g for 5 minutes to pellet the cellular material. The tagmentation process was executed in 1 × Tagmentation Buffer that included 10 mM Tris at pH 7.4, 5 mM MgCl2, 10% DMF, 33% PBS, 0.1% TWEEN 20, and 0.01% Digitonin, employing 100 nM of Tn5 Transposase for 30 minutes at 37°C. Immediately after tagmentation, the free DNA fragments were purified following the protocol specified by the QIAquick PCR Purification Kit (QIAGEN, 28106, Germany). This step was followed by a final PCR amplification cycle of 10 to 15 rounds using P5 and P7 primers. Post-purification, the prepared libraries were sequenced via the Illumina NovaSeq 6000 sequencing platform to generate 150bp pair-end reads.

### 2.7 LAM-HTGTS

LAM-HTGTS was performed using 1 μg DNA of sorted DN cells or 6 μg DNA of sorted DP cells from one WT or EACBE^-/-^ mice per experiment. DNA was extracted using DNA Isolation Mini Kit (Vazyme, DC102) and sonicated to about 500bp on a Qsonica Bioruptor Sonicator. Sonicated DNA was linearly amplified with a biotinylated primer that anneals to sites of interest. Biotin-labeled single stranded DNA products were enriched with streptavidin C1 beads (65001, Thermo Fisher Scientific), and followed by 3’ end ligation with the bridge adapter. The adapter-ligated products were amplified through nested PCR using a nested primer and an adapter-complementary primer (**Supplementary Table S3**). The detailed primers used in this study are also listed in **Supplementary Information**, **Supplementary Table S3**. And a final PCR for another 10–12 cycles of amplification with P5 and P7 primers was performed. After purification, libraries were sequenced on an Illumina NovaSeq 6000 platform to obtain 150 bp pair-end reads.

### 2.8 Germline transcription for qPCR or RNA-Seq

RNA was extracted from DN thymocytes from *Rag1* ^-/-^ or EACBE^-/-^ *Rag1* ^-/-^ mice employing TRIzol reagent (Invitrogen), adhering strictly to the manufacturer’s protocol. 500ng RNA was used to synthesize cDNA according to the manufacturer’s instructions (Vazyme, R312). Quantitative real-time PCR (qPCR) was then conducted utilizing a Relative Quantification approach. The thermal cycling conditions were set as follows: an initial denaturation at 95°C for 5 minutes, followed by 45 cycles of denaturation at 95°C for 30 seconds, and annealing/extension at 60°C for 1 minute. The primer sequences employed are cataloged in **Supplementary Tables S4** or **S5**. The relative expression levels of various gene transcripts were computed using the comparative ΔΔCt method, where the ΔΔCt value for each target gene was normalized against that of the housekeeping gene Actb.

For subsequent library construction, 1μg of total RNA was processed. Initially, ribosomal RNA (rRNA) was removed using an rRNA depletion kit, and the remaining mRNA was fragmented into shorter segments (200–300 bp) with the addition of a fragmentation buffer. First-strand cDNA synthesis was initiated using random hexamer primers, while second-strand cDNA was synthesized in the presence of buffer, deoxynucleotide triphosphates (dNTPs including dUTP, dATP, dGTP, and dCTP), RNase H, and DNA polymerase I. The cDNA was subsequently purified using the QiaQuick PCR kit and eluted with EB buffer. Following this, the cDNA underwent end repair, adenylation, and ligation with Illumina adapters. The second cDNA strand containing uracil was specifically degraded by the USER enzyme. Lastly, PCR amplification was performed to enrich for strand-specific cDNA libraries. Post-purification, these libraries were subjected to high-throughput sequencing on the Illumina NovaSeq 6000 platform, generating 150bp paired-end reads.

### 2.9 Native chromatin immunoprecipitation-qPCR

Native ChIP was performed on DN thymocytes from 3–4 *Rag2* ^-/-^ or EACBE^-/-^ *Rag2* ^-/-^ mice per experiment. Cells were lysed in 200μl of a buffer containing 80 mM NaCl, 10 mM Tris-HCl pH8.0, 10 mM sodium butyrate, 6 mM MgCl_2_, 1 mM CaCl_2_, 250 mM sucrose, 0.2% (vol/vol) NP40, 0.1 mM PMSF, and 1×protease inhibitor cocktail, followed by a 5-minute incubation on ice. The lysate was then subjected to centrifugation at 600 × g for 5 minutes at 4°C. The nuclear pellet was subsequently washed once with a buffer composed of 10 mM NaCl, 10 mM Tris-HCl (pH 8.0), 10 mM sodium butyrate, 3 mM MgCl_2_, 1 mM CaCl_2_, and 250 mM sucrose. To generate predominantly mononucleosomes with a minor fraction of dinucleosomes, the nuclei were digested by incubating them for 5 minutes at 37°C in 200 μl of the same buffer supplemented with 8 units of Micrococcal nuclease (Worthington). The enzymatic reaction was halted by adding 8 μl of a stop solution containing 0.2 M EDTA and 0.2 M EGTA. Following centrifugation at 18,000 × g for 10 minutes, the supernatant was diluted to achieve a final concentration of 16.7 mM Tris (pH 8.0), 1.2 mM EDTA, 167 mM NaCl, 1.1% Triton X-100 (v/v), 0.1 mM PMSF, and 1× protease inhibitor cocktail. The chromatin was then incubated overnight at 4°C with specific antibodies: anti-trimethylated H3K4 (Millipore, 04-745), anti-acetylated H3K27 (Abcam, ab4729), or control rabbit IgG (R&D Systems, ab-105-c). Protein A/G magnetic beads (Pierce, 88802) were added to the mixture and incubated for an additional four hours. Post-incubation, the immunoprecipitates were rigorously washed, and the DNA was purified for subsequent analysis.

Quantitative PCR (qPCR) was performed using a StepOne™ Real-Time PCR System (Thermo Fisher, 4376373) with Hieff™ qPCR SYBR^®^ Green Master Mix (YEASEN, China). A standard curve was constructed using gradient concentrations of genomic DNA to ensure accurate quantification. Both immunoprecipitated and input DNAs were quantified, and the Actb gene promoter served as a positive control to normalize the bound/input ratios across different samples. Detailed primer sequences are provided in **Supplementary Table S6**. The PCR protocol included an initial denaturation step at 95°C for 5 minutes, followed by 45 cycles of 30 seconds at 95°C and 1 minute at 60°C.

### 2.10 3C-HTGTS data processing for pairwise chromatin interactions

Paired-end Illumina sequencing FASTQ data were processed by removing adapters and low-quality reads using Fastp (v0.20.0). Following quality control, trimmed reads were extracted from the sequence files with Cutadapt (v1.18). Paired-end reads containing nested primers or adapter primers were manually merged into single reads using restriction enzyme recognition sequences with PEAR (v0.9.6). Subsequently, the initial digested fragment located behind the viewpoint (VP) was isolated by fragmenting the single reads according to restriction enzyme recognition sequences. The remaining single-end reads were aligned to the enzyme-digested mm10 reference genome using Bowtie2 (v2.4.5, parameters: -p 8 –sensitive). The mouse genome sequence (mm10) was sourced from UCSC (http://hgdownload.cse.ucsc.edu/goldenPath/mm10/bigZips/chromFa.tar.gz), and concordantly exact alignments were extracted using SAMtools (v1.9). Self-ligation and off-target reads were filtered out post-mapping. For visualization purposes, the final BAM files were converted into bedGraph files using Bedtools (v2.29.2). The signal peak bedGraph file was generated through a process of post-comparison filtering, signal statistical analysis, and standardization. We applied the CPM (Counts Per Million in cis) normalization method to the bedGraph files and visualized the results using the IGV genome browser. Differential pairwise interactions were identified using the R package R.4Cker (version 1.0.0, with k=30), employing the near viewpoint Analysis function to delineate interaction domains with the viewpoint. Additionally, DESeq2 (version 1.34.0, with a significance threshold of p < 0.05) was utilized for further analysis (32). Finally, we compiled the results into a comprehensive report and visualized the data using the Bioconductor package ggplot2 (version 3.3.6).

### 2.11 3C-HTGTS data processing for three-way chromatin interactions

The quality control of the raw data and the fragmentation process based on restriction enzyme sites were conducted in accordance with the previously described pairwise chromatin interaction method. Subsequently, all fragments retrieved from the same read were organized on a single line according to the unique identifier of each read, and continuous fragments were removed. To construct contact matrices, the first two digested fragments following the viewpoint fragment were extracted, or various combinations of three fragments were generated by arranging all fragments from the same read. Raw contact matrices were produced at resolutions of 3 kb, 5 kb, and 10 kb. For the correction of raw contact matrices, these interaction counts were normalized to a total of 1,000,000 interactions at the same resolutions. Like a Hi-C matrix, coverage was represented in a two-dimensional matrix, where each point indicated the number of interactions identified between two bins at a specific resolution. Differential analysis and visualization of local interactions derived from three-way interactions were performed using the R package GENOVA (v1.0.0). Loops observed on the IGV genome browser were identified using fixed-size bin resolutions ranging from 3 kb to 10 kb. Briefly, interaction loops (contact frequencies >= 5) were identified by using raw contact frequencies.

### 2.12 VP-SOI analysis for three-way interactions for 3C-HTGTS

In accordance with the methodology described by Vermeulen et al. (33), our study identified cooperative, random, or competitive multi-way interactions involving the viewpoint (VP) and two additional sites of interest: a second site of interest (SOI) and a third site. This was achieved through an association analysis. Specifically, in cases where the interaction is cooperative among the VP, SOI, and the third site, a subset of reads containing both the VP and SOI should also frequently encompass the third site. To evaluate whether the third site exhibits cooperative, random, or competitive interactions, we compared its frequency in the set of reads containing both the VP and SOI (referred to as the positive set) with its frequency in the set of reads containing the VP but lacking the SOI (referred to as the negative set). To mitigate the effects of technical and sampling variations, we randomly sampled same reads from the negative set equivalent to the number of reads in the positive set. Subsequently, we randomly filtered one fragment from each sampled read in the negative set to substitute for the SOI fragment present in all reads of the positive set. This procedure was iterated 1,000 times to construct an average negative profile, with the mean and standard deviation calculated accordingly. Subsequently, the positive contact profile was compared to the negative profile, and a z-score was computed to assess the significance of cooperative or competitive interactions among the VP, the SOI, and the third partner. A z-score approaching zero suggests a random contact frequency between the SOI and the third partner in the presence of the VP, whereas a positive or negative z-score indicates cooperative or competitive interactions among these three genomic regions, respectively.

### 2.13 ATAC-Seq analysis

The raw sequence reads were initially processed to remove adapter sequences and low-quality reads using fastp (version 0.20.0). Subsequently, the filtered reads were aligned to the mouse genome (mm10) utilizing Bowtie2 (version 2.4.5) with parameters set to -p 8 –sensitive. PCR duplicate fragments were removed using Picard (version 2.22.8). Unmapped, multi-mapped reads, as well as those mapping to chromosome M (chrM), were filtered out. The Fragments Ratio in Peaks (FRiP) value was calculated using Bedtools (version 2.29.2) and awk (version 4.0.2). We employed deepTools (version 3.5.0) to generate bigWig files with CPM normalization, which can be visualized in IGV. SAM files were converted to BAM format using SAMtools (version 1.9) for subsequent peak calling. Peaks were identified using MACS2 (version 2.2.4) with specified parameters (–nomodel –shift -100 –extsize 200 -B –keep-dup all –broad –broad-cutoff 0.1), and annotations were performed using the R package ChIPSeeker (version 1.36.0).

### 2.14 *Tcra* repertoire analysis

*Tcra* repertoire sequencing data were obtained from our publicly available resource. The detailed analytical methodology has been previously described (28). To determine the differences in Vα gene usage, the usage of each Vα gene in EACBE^-/-^ was subtracted from its corresponding usage in the WT.

### 2.15 LAM-HTGTS analysis

The initial raw data underwent filtration using fastp (version 0.20.0). Subsequently, trimmed reads, which included nested primers and adapter primers, were removed and extracted from the sequence file following quality control procedures implemented with Cutadapt (version 1.18). Additionally, reads exhibiting contamination or low quality were eliminated. The identification of T-cell receptor alpha and delta chain V, D, and J genes, as well as the extraction of CDR3 sequences from the clean reads, was conducted utilizing MiXCR (version 3.0.11) (available at https://github.com/milaboratory/mixcr). The corresponding germline sequences were aligned with reference sequences obtained from the international ImMunoGeneTics (IMGT) database.

## 3 Results

### 3.1 EACBE promotes proximal V_δ_ usage while restricting distal V_δ_ rearrangement

In our previous study, we analyzed the *Tcrd* repertoire in wild-type (WT) and EACBE^-/-^ mouse using 5’ rapid amplification of cDNA ends (5’ RACE) with a C_δ_-specific primer. The results revealed a significant reduction in the usage of proximal V_δ_ segments, such as *Trdv2–2* and *Trdv1*, in EACBE-deleted thymocytes, whereas the usage of *Trdv5* and distal V_δ_ segments was increased (28). These findings suggest that EACBE contributes to the regulation of V(D)J recombination of the *Tcrd* gene. The enhancer E_α_ was previously shown to be dispensable for *Tcrd* rearrangement but necessary for maintaining physiological expression levels of mature VDJ_δ_ transcripts (30). To determine whether EACBE directly regulates *Tcrd* rearrangement, we performed a PCR-Southern blot assay. The results confirmed that EACBE deletion leads to an increase in *Trdv5* rearrangements and a decrease in *Trdv2–2* rearrangements (**Supplementary Figure S1A**).

**Figure 1:**
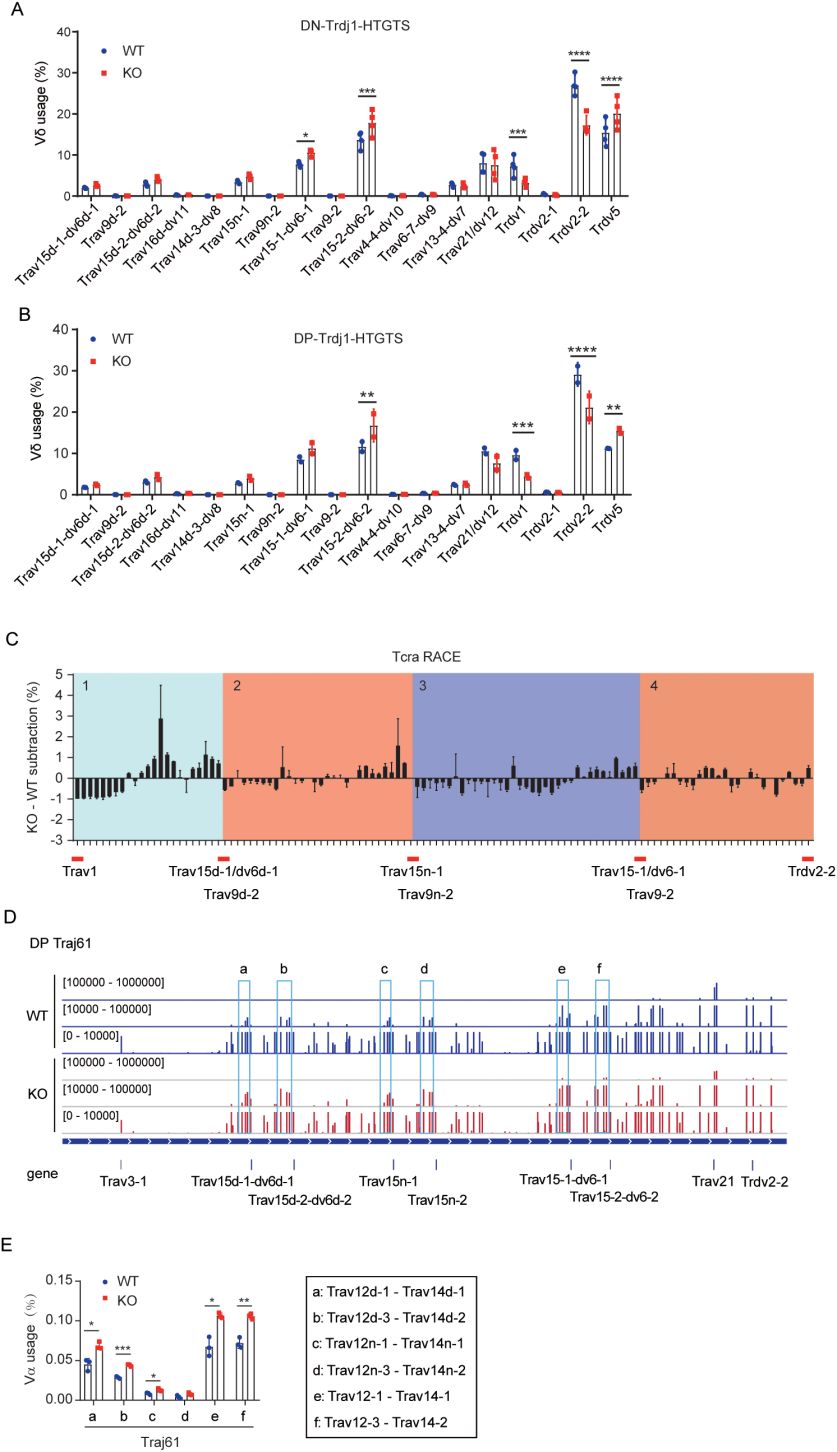
EACBE regulates V_α_ gene usage by modulating the rearrangement of distal V_δ_ segments. **(A)** The usage of V_δ_ segments was detected by LAM-HTGTS from the *Trdj1* viewpoint in sorted DN thymocytes from WT (Blue circle) and EACBE^-/-^ (Red quadrate) mice. Data represent the mean ± s.d. of four experiments. *P <0.05, ***P <0.001, ****P <0.0001 by two-side multiple Student’s *T* test. **(B)** The usage of V_δ_ segments was detected by LAM-HTGTS from the *Trdj1* viewpoint in sorted DP thymocytes from WT (Blue circle) and EACBE^-/-^ (Red quadrate) mice. Data represent the mean ± s.d. of two experiments. **P <0.01, ***P <0.001, ****P <0.0001 by two-side multiple Student’s *T* test. **(C)** EACBE^-/-^ to WT subtraction of V_α_ usage, calculated from previous *Tcra* repertoire sequencing data (GEO: GSE145147). **(D)** Detection of V_α_ peaks by LAM-HTGTS with the *Traj61* viewpoint in sorted DP thymocytes from WT (Blue) and EACBE^-/-^ (Red) mice. Each experiment was repeated three times. The Y-axis represents the binding strength of the peaks that are rearranged with VP. **(E)** Histogram showing the usage of three V_α_ segments located behind the Trav15 family from panel **(D)**. Data represent the mean ± s.d. of three experiments. *P <0.05, **P <0.01, ***P <0.001 by two side multiple Student’s *T* test.

To obtain a more comprehensive view of *Tcrd* rearrangement dynamics, we employed Trdj1-HTGTS-seq to examine the usage of V_δ_ genes in sorted DN cells. In WT cells, frequently used V_δ_ segments included *Trdv5*, *Trdv2-2*, *Trdv1*, *Trav21*/*dv12*, *Trav15-2*/*dv6-2*, *Trav15-1*/*dv6-1*, *Trav15n-1*, *Trav15d-2*/*dv6d-2*, and *Trav15d*/*dv6d-1*, with *Trdv2–2* being the most prominently used. Compared to WT DN cells, the usage of *Trdv5* and distal Trav15 family genes was increased in EACBE-deleted DN cells, alongside reduced usage of proximal V_δ_ genes (*Trdv2–2* and *Trdv1*) (**Figure 1A**), which is consistent with the 5’ RACE results. These data support a direct regulatory role of EACBE in *Tcrd* rearrangement.

To assess the functional consequences of altered *Tcrd* rearrangement, we assessed the γδ T cell populations in the thymus, spleen, and lymph nodes of WT and EACBE^-/-^ mice. Although γδ T cell proportion were comparable in the thymus and spleen, a slight reduction was observed in the lymph nodes of EACBE^-/-^ mice (**Supplementary Figure S1B**). These results suggest that EACBE deletion does not markedly impair γδ T cell development.

Previous studies have demonstrated that *Tcrd* rearrangement increases the usage of V_α_ segments in the repeat region and enhances the diversity of the *Tcra* repertoire (26, 34). Therefore, we examined the usage of V_δ_ genes in sorted DP thymocytes from WT and EACBE^-/-^ mice using Trdj1-HTGTS-seq. We observed that the V_δ_ usage profile in WT DP cells mirrored that in WT DN cells. In contrast, EACBE^-/-^ DP cells exhibited increased rearrangement of *Trdv5* and distal V_δ_ genes and reduced rearrangements of proximal V_δ_ segments (**Figure 1B**). These findings indicate that EACBE may indirectly regulates *Tcra* rearrangement by modulating *Tcrd* rearrangement during the DN stage.

### 3.2 EACBE restricts Trav15 family rearrangements at the DN stage

Our recent study demonstrated that EACBE deletion changed V_α_ usages in *Tcra* primary rearrangement (28). To assess whether this change is attributable to alterations in *Tcrd* rearrangement in EACBE-deficient mice, we performed a comprehensive analysis of V_α_ usage in WT and EACBE^-/-^ thymocytes, utilizing previous *Tcra* 5’ RACE data. The analysis revealed a distinctive, repetitive alteration in V_α_ usage, in which V_α_ segments could be grouped into four repetitive domains based on three frequently used V_δ_ segments from the Trav15 family: 1) *Trav1* to *Trav15d-1/dv6d-1*, 2) *Trav9d-2* to *Trav15n-1*, 3) *Trav9n-2* to *Trav15-1/dv6-1*, and 4) *Trav9–2* to *Trdv2-2* (**Figure 1C**). Notably, the overall V_α_ usage within these four regions remained unchanged in EACBE^-/-^ mouse thymocytes compared to WT (**Supplementary Figure S1C**). However, within region 4, the usage of certain proximal V_α_ genes increased. Furthermore, EACBE deletion resulted in increased usage of several V_α_ segments just upstream of the Trav15 family members in the other three regions, followed by a subsequent decrease in usage from 3’ to 5’ regions (**Figure 1C**, **Supplementary Figure S1D**). Consequently, we hypothesized that EACBE modulates V_α_ gene usage by regulating distal V_δ_ usage, especially the Trav15 family.

To confirm this, we detected *Tcra* primary rearrangements in sorted DP cells from WT and EACBE^-/-^ mice using *Traj61*-HTGTS-seq. Although *Traj61* is a pseudogene, it is the first J_α_ gene to undergo rearrangement, and its rearrangement serves as a marker for *Tcra* primary rearrangement. In WT DP cells, *Traj61* predominantly rearranged with proximal V_α_ genes, particularly *Trav21*, consistent with previous findings (28, 34). Additionally, we observed frequent rearrangements of *Traj61* with V_α_ segments just upstream of the Trav15 family (**Figure 1D**). In contrast, EACBE deletion in DP cells resulted in a reduction of *Traj61* rearrangements with proximal V_α_ genes, like *Trav21*, while increasing rearrangements with the *Trav12*-*Trav14* region upstream of the Trav15 family (**Figures 1D, E**). These findings suggest that EACBE not only facilitates proximal V_α_-to-J_α_ rearrangements during the DP stage but also restricts Trav15 family rearrangements during the DN stage, thereby preserving the diversity of the *Tcra* repertoire at the DP stage. Recent work by Danielle J et al. (27) revealed that the usage of the Trav15-dv6 family in *Tcrd* recombination enhances *Tcra* repertoire diversity, further supporting our observations.

### 3.3 EACBE influences Trav14 family rearrangements during the DP stage by modulating Trav15 family rearrangements during the DN stage

**Figure 2:**
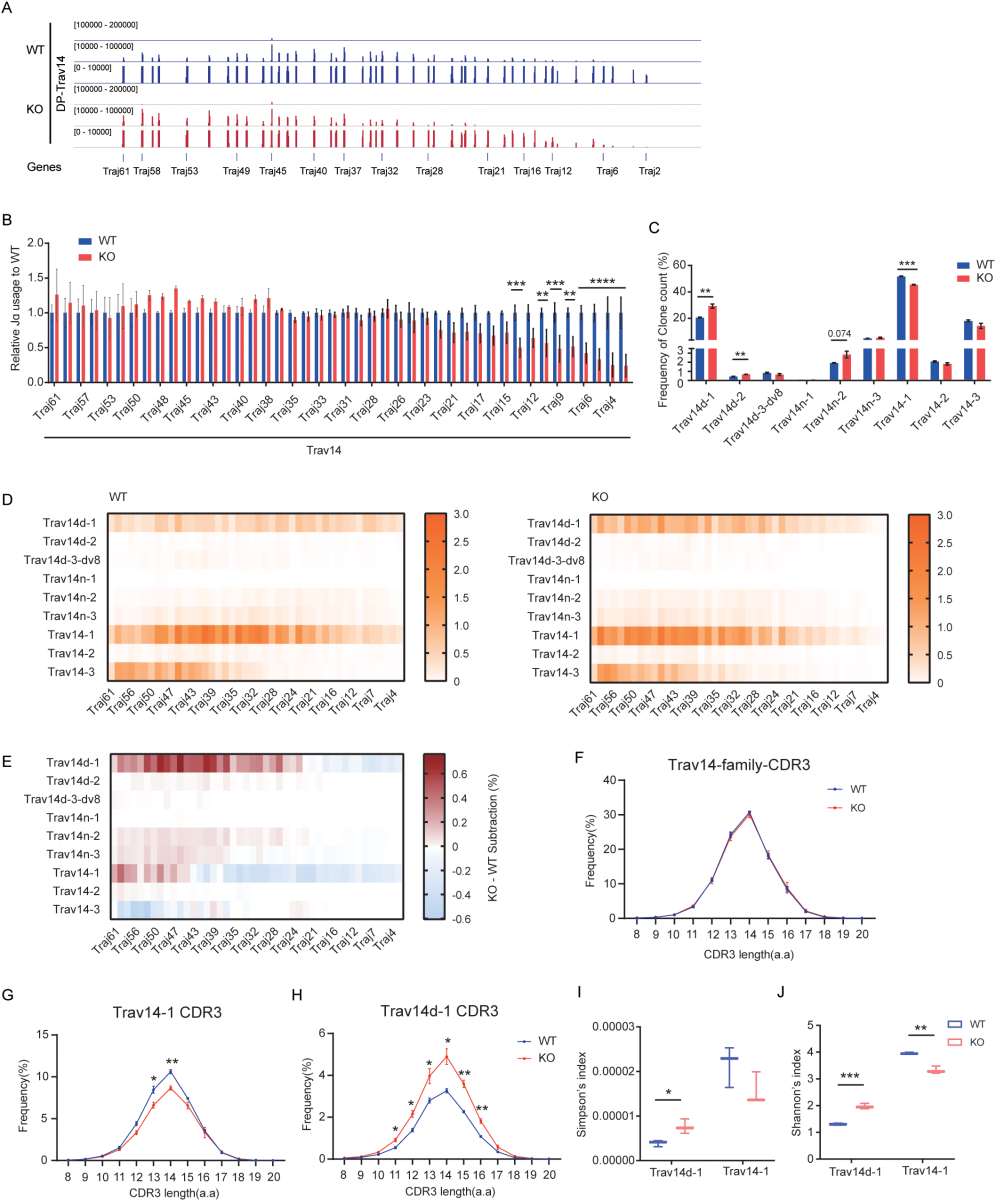
EACBE regulates the diversity of *Trav14* family-related TCRs. **(A)** Detection of J_α_ peaks by LAM-HTGTS with the *Trav14* viewpoint in sorted DP thymocytes from WT (Blue) and EACBE^-/-^ (Red) mice. Each experiment was repeated three times. The Y-axis represents the binding strength of the peaks that are rearranged with VP. **(B)** Histogram showing relative J_α_ usage of from panel **(A)**. Data represent the mean ± s.d. of three experiments. **P <0.01, ***P <0.001 by two side multiple Student’s *T* test. **(C)** Histogram showing the usage frequency of each member of the Trav14 family from panel **(A)**. Data represent the mean ± s.d. of three experiments. **P <0.01, ***P <0.001, ****P <0.0001 by two side multiple Student’s *T* test. **(D)** Heatmap showing the *Trav14*-Jα combination of each Trav14 member in sorted DP thymocytes from WT and EACBE^-/-^ mice. Each experiment was repeated three times. **(E)** Heatmap of EACBE^-/-^ – WT subtraction represents the *Trav14*-J_α_ combination of the differences from panel **(D)**. Data represent the mean of three experiments. **(F)** CDR3 lengths of Trav14 family repertoires in WT and EACBE^-/-^ mice. Data represent the mean ± s.d. of three experiments. **(G, H)** CDR3 length of *Trαv14-1* **(G)** and *Trαv14d-1* **(H)** repertoires in WT and EACBE^-/-^ mice. Data represent the mean ± s.d. of three experiments. *P <0.05, **P <0.01 by two side multiple Student’s *T* test. **(I, J)** Simpson’s index **(I)** and Shannon’s index **(J)** of *Trαv14–1* and *Trαv14d-1* repertoires in WT and EACBE^-/-^ mice. Data represent the mean ± s.d. of three experiments.

Sleckman BP et al. reported that the usage of V_α_ segments in peripheral T cells of E_α_-deficient mice were highly restricted, as the majority of these cells expressed Trav14(V_α_2)-related TCRs, compared to 5%–10% of peripheral T cells in WT mice (30). To elucidate the effect of EACBE on Trav14 rearrangement, we analyzed Trav14 family rearrangements in sorted DP cells from WT and EACBE-deficient mice using Trav14-HTGTS sequencing. The results revealed a significant increase in rearrangement between the Trav14 family and 5’ J_α_ segments (from *Traj61* to *Traj38*), and a significant decrease in rearrangement with 3’ J_α_ segments (*Traj21* to *Traj2*) (**Figures 2A, B**), consistent with 5’ RACE results.

The Trav14-HTGTS sequencing panel contains nine V_α_ members of the Trav14 family, located at varying distances from the *Tcra* gene recombination center, namely *Trav14d-1*, *Trav14d-2*, *Trav14d-3-dv8*, *Trav14n-1*, *Trav14n-2*, *Trav14n-3*, *Trav14-1*, *Trav14-2*, and *Trav14-3* (2). In WT mice, *Trav14d-1*, *Trav14-1*, and *Trav14–3* are the most frequently used segments, with *Trav14–1* being the most prevalent (**Figure 2C**, **Supplementary Figure S2A**). The frequencies of segments within the Trav14 family do not align consistently with those observed in the Trav15 family. For instance, *Trav14–2* is located upstream of *Trav15-2-dv2*, which demonstrates the highest rearrangement frequency among Trav15 family members. The rearrangement of the Trav15 family with DJ_δ_ in DN cells reduces the spatial distance between Trav14 family members and the *Tcra* recombination center in DP cells. Despite *Trav14–2* exhibiting the highest primary rearrangement frequency within the Trav14 family, its overall usage frequency remains relatively low compared to other Trav14 family members (**Figures 1A, E**, **2C**). EACBE deletion significantly enhances the rearrangement of *Trav14d-1* and *Trav14d-2*, while significantly reduces the rearrangement of *Trav14-1* (**Figure 2C**, **Supplementary Figure S2A**). Furthermore, we observed a significant increase in the rearrangement of each Trav14 member with 5’ J_α_, except for *Trav14-3* (**Figures 2D, E**). These results suggest that EACBE plays a role in modulating the diversity of Trav14-related TCRα chain.

The CDR3 region of the antigen receptor is crucial for antigen recognition, with its amino acid composition playing a central role in determining specificity (35). To assess the impact of EACBE on CDR3 diversity, we conducted an analysis of the amino acid sequence characteristics of CDR3 in Trav14-related T-cell receptor α (TCRα). The result revealed that EACBE deletion did not alter the amino acid length or composition of CDR3 within the Trav14 family (**Figure 2F**, **Supplementary Figure S2B**). However, it did influence the frequency distribution of CDR3 lengths and types among various members of the Trav14 family (**Figures 2G, H**, **Supplementary Figures S2C, 2D**). Additionally, we observed a slight, albeit statistically insignificant, reduction in the overall CDR3 diversity of the Trav14 family following EACBE deletion (**Supplementary Figures S2E, 2F**). Interestingly, the CDR3 diversity of individual Trav14 family members either increased or decreased, consistent with the rearrangement outcomes (**Figures 2I, J**). These results indicate that EACBE plays a regulatory role in the rearrangement processes and diversity of TCRs associated with the Trav14 family.

### 3.4 EACBE deletion reduces chromatin activity at the *Trdv2–2* site

**Figure 3:**
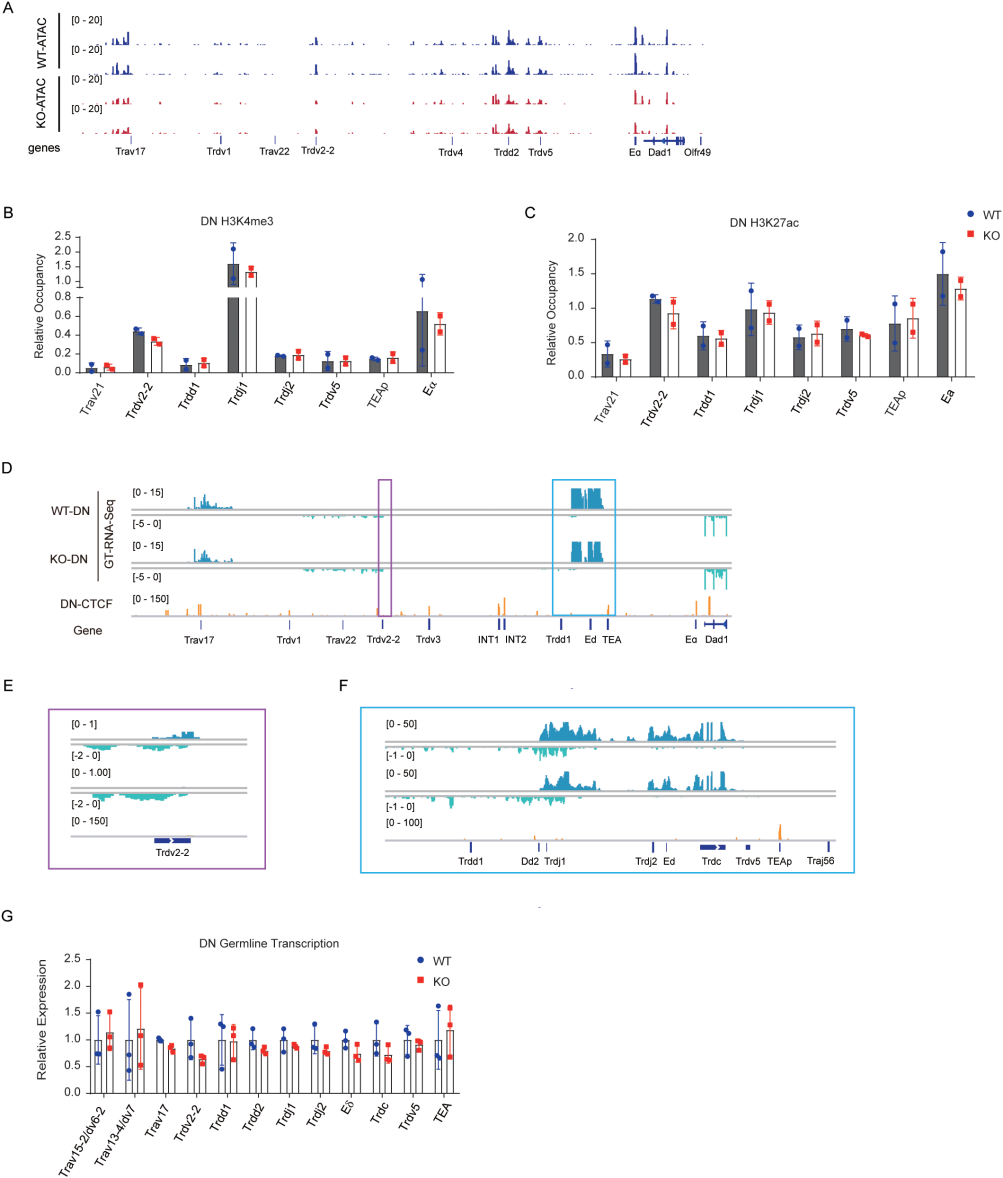
EACBE regulates chromatin activity of Tcrd gene in DN cells. **(A)** ATAC-seq signals on the *Tcra*-*Tcrd* locus in DN thymocytes from *Rag1* ^−/−^ and EACBE^−/−^ *Rag1* ^−/−^ mice. Data were representative of two independent experiments. **(B, C)** Histone H3K4me3 **(B)** and H3K27ac **(C)** modification analyzed by ChIP-qPCR on the *Tcra*-*Tcrd* locus in DN thymocytes from *Rag2* ^−/−^ and EACBE^−/−^ *Rag2* ^−/−^ mice. Each experiment was repeated twice. **(D-F)** Genome browser views depicting GT-RNA-seq data of the *Tcra-Tcrd* locus in DN thymocytes from *Rag1* ^−/−^ and EACBE^−/−^ *Rag1* ^−/−^ mice. Positive strand transcription is shown in Ocean Blue, and negative strand transcription is shown in pale green. Coordinates (mm10): chr14: 53747480-54261865. Rectangles of the same color correspond to the same enlarged area. Data represent the one experiment. **(G)** Relative germline transcription in the *Tcra*-*Tcrd* locus in DN thymocytes from *Rag1* ^−/−^ and EACBE^−/−^ *Rag1* ^−/−^ mice detected using reverse-transcription qPCR. Expressions were normalized to the *Actb* gene. Data represent the mean ± s.d. of three experiments.

The accessibility and germline transcription of antigen receptor genes are crucial for regulating V(D)J recombination (36). To investigate whether EACBE modulates *Tcrd* rearrangement by influencing the chromatin activity of the *Tcrd* locus, we assessed accessibility, active histone modifications, and germline transcription at the *Tcra*-*Tcrd* locus in DN cells. ATAC-seq analysis revealed a marked decrease in the accessibility of *Trdv5*, *Trdd2*, *Trdv2-2*, and upstream region of Trav17 (**Figure 3A**). Chromatin markers indicative of active regions, such as H3K27 acetylation (H3K27ac) (37) and H3K4 trimethylation (H3K4me3) (38–40), are integral to V(D)J recombination. ChIP-qPCR assays showed no statistically significant alterations in these active chromatin marks across most regions of the *Tcra*-*Tcrd* locus following EACBE deletion, including *Trav21*, *Trdd1*, *Trdj1*, *Trdj2*, *Trdv5*, TEAp, and E_α_. Specifically, at the *Trdv2–2* promoter, we noted slight reductions in H3K4me3 and H3K27ac levels (**Figures 3B, C**). These results suggest a potential impact on the transcriptional activity of *Trdv2-2*.

To further explore the effects of EACBE deletion on *Tcrd* transcriptional activity, we conducted GT-RNA-Seq using DN thymocytes derived from EACBE^+/+^ × Rag1^−/−^ and EACBE^−/−^ × Rag1^−/−^ mice. In WT DN cells, the highest transcriptional activity was observed at the *Tcrd* recombination center, followed by a region proximal to *Trav17*. Weak transcriptional activity was also detected in the region between *Trdv1* and *Trdv2-2* (**Figure 3D**). These transcriptional patterns align with the rearrangement activity of *Tcrd* in DN cells. EACBE deletion resulted in a reduction of forward transcription at *Trdv2-2*, while reverse transcription experienced a slight increase (**Figure 3E**). Furthermore, transcription at D_δ_-J_δ_ segments were modestly decreased in EACBE-deficient mice (**Figure 3F**).

To corroborate these findings, RT-qPCR experiments were conducted to assess germline transcription of *Tcrd* gene. Although the results did not reach statistical significance, EACBE deletion was associated with a reduction in the germline transcription of these segments, including *Trav17*, *Trdv2-2*, *Trdd2*, E_δ_, and *Trdc*, with *Trdv2–2* exhibiting the most pronounced decrease (**Figure 3G**). These findings suggest that EACBE deletion attenuated chromatin activity at the *Trdv2–2* promoter, leading to a subsequent decrease in its rearrangement.

### 3.5 EACBE regulates the spatial organization of the *Tcrα-Tcrd* locus at the DN stage

**Figure 4:**
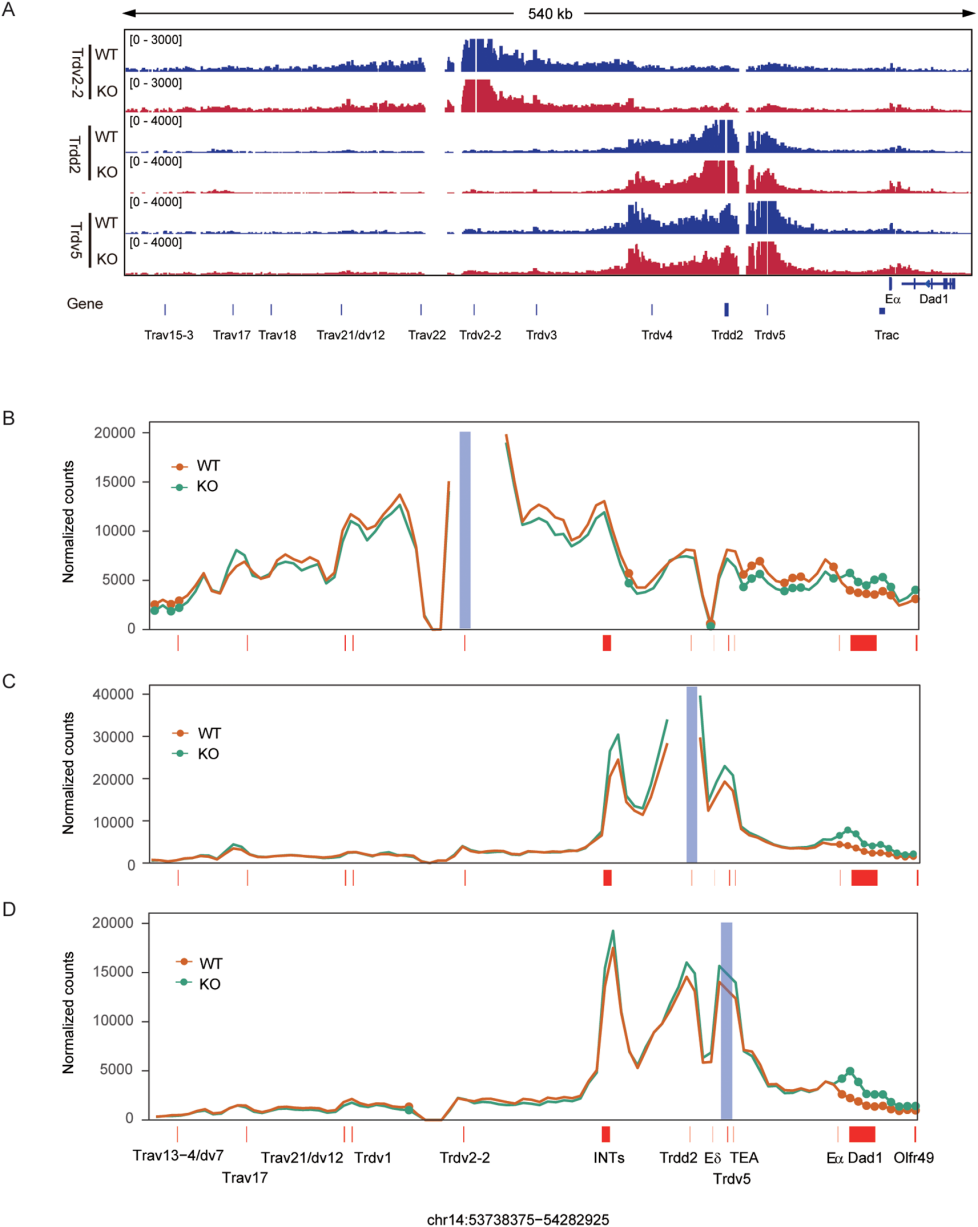
EACBE regulates the spatial organization of the *Tcrα-Tcrd* locus at the DN stage. **(A)** Genome browser views depicting 3C-HTGTS pairwise chromatin interactions from *Trdv2-2*, *Trdd2*, and *Trdv5* viewpoints in the 3’ portion of the *Tcra*-*Tcrd* locus in DN thymocytes from *Rag1* ^−/−^ (WT, blue) and EACBE^−/−^ *Rag1* ^−/−^ (KO, red) mice. 3C-HTGTS is representative of three replicates for each viewpoint. Gene annotations are shown below. Coordinates (mm10): chr14: 53738375-54282925. **(B-D)** Line plots displaying the difference of pairwise interactions between *Rag1* ^−/−^ (WT, orange) and EACBE^−/−^ *Rag1* ^−/−^(KO, green) mice at the *Trdv2-2* **(B)**, *Trdd2* **(C)** and *Trdv5* **(D)** viewpoints using the 4C-ker program. Analysis is based on three independent experimental replicates. Filled circles highlight significant differential interactions (*P* < 0.05; statistics derived using DESeq2). Gene positions are annotated by red-filled rectangles and the blue-filled bar highlights the viewpoint position.

We previously reported that EACBE deletion reduced interactions between the proximal V_α_ and proximal J_α_ regions in DP thymocytes (28). To further explore the effect of EACBE deletion on interactions involving *Trdv2-2*, *Trdv5*, and *Trdd2*, we conducted a 3C-HTGTS assay using these segments as viewpoints. In DN cells from Rag1-deficient mice, *Trdv2–2* exhibited substantial interactions with sequences extending from upstream *Trav21* to downstream INTs (**Figures 4A, B**). Notably, EACBE deletion resulted in a significant reduction in interactions between *Trdv2–2* and sequences from INTs to E_α_, including the D_δ_-J_δ_-C_δ_ region (**Figure 4B**). Additionally, we observed a significant increase in interactions between *Trdv2–2* and the downstream region of EACBE, which may be attributed to the EACBE deletion weakening the insulation at the TAD boundary in which it is situated (**Supplementary Figures S3A**, **S3B**).

Interactions involving *Trdd2* were confined to the region between INTs and TEAp (**Figures 4A, C**), consistent with previous observation (26). As anticipated, the deletion of EACBE resulted in increased interactions of *Trdd2* with sequences located downstream of EACBE (**Supplementary Figures S3A**, **S3C**). The deletion also increased interactions between *Trdd2* and sequences between INTs and TEAp, including *Trdv5*, while leaving interactions with sequences upstream of INTs unaffected (**Figure 4C**). Furthermore, *Trdv5* exhibited slightly increased interactions with sequences between INTs and TEA (**Figures 4A, D**), consistent with *Trdd2* 3C-HTGTS data. *Trdv5* also demonstrated increased interactions with sequences downstream of EACBE (**Supplementary Figures S3A**, **S3D**). Our previous research demonstrated that the EACBE deletion would affect the expression of its downstream genes in the thymocyte cells (28). However, the EACBE deletion did not exhibit a similar impact on downstream gene expression in DN cells (**Supplementary Figure S3E**).

In summary, these results indicate that EACBE establishes a TAD boundary at the DN stage to restrict the interaction between the *Tcra*-*Tcrd* locus and its downstream regions. We also observed that EACBE deletion not only enhanced interactions within the region from INTs to TEAp, but also weakened interactions between *Trdv2–2* and the region from INTs to E_α_. This suggests that EACBE deletion enhance the insulation of INTs. In brief, EACBE is involved in regulating the spatial organization of the *Tcrα-Tcrd* locus at the DN stage, facilitating the normal rearrangement of the *Tcrα-Tcrd* locus.

### 3.6 EACBE reduces the insulation of INTs in DN cells

**Figure 5:**
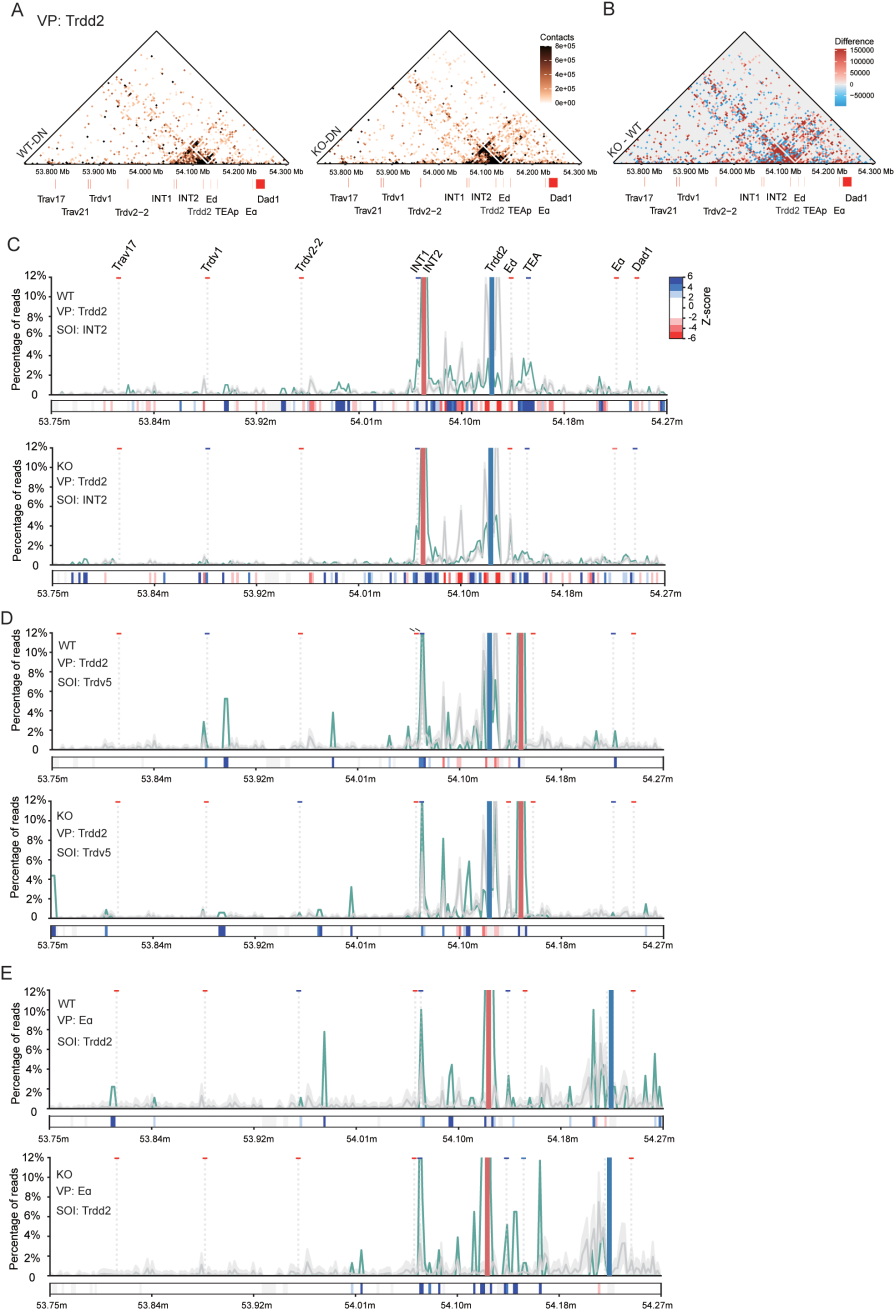
Effect of EACBE on higher-order chromatin structure of *Tcrα-Tcrd* locus in DN cells. **(A)** Heatmap showing three-way chromatin interactions in the 3’ portion of the Tcrα-Tcrd locus from the Trdd2 viewpoint in DN thymocytes from *Rag1* ^−/−^ (WT, up) and EACBE^−/−^ *Rag1* ^−/−^(KO, down) mice. The heatmap represents mean of three experimental replicates. Gene annotations are shown middle. Resolution: 5kb; Coordinates (mm10): chr14: 53738375-54282925. **(B)** EACBE^-/-^ – WT subtraction heatmap (resolution: 5kb) showing the three-way contact differences from panel **(A)**. **(C)** VP-SOI plots displaying co-occurrence contacts of sequences in the 3’ portion of the Tcra-Tcrd locus in the combination of the Trdd2 viewpoint (pale blue rectangle) and the SOI containing INT2 (pale red rectangle) in DN thymocytes from *Rag1* ^−/−^ and EACBE^−/−^ *Rag1* ^−/−^ mice. The green line represents the observed co-occurrence frequency, and the gray line represents the expected frequency (mean ± s.d.) of sequences across the locus. z-scores (dark blue indicating significant enrichment, dark red indicating significant lack of a given site) are shown for SOIs in rectangles below each graph. Gene annotations are at the top. **(D)** VP-SOI plots displaying co-occurrence contacts of sequences in the 3’ portion of the Tcra-Tcrd locus in the combination of the Trdd2 viewpoint (pale blue rectangle) and the SOI containing *Trdv5* (pale red rectangle) in DN thymocytes from *Rag1* ^−/−^ and EACBE^−/−^ *Rag1* ^−/−^ mice. **(E)** VP-SOI plots displaying co-occurrence contacts of sequences in the 3’ portion of the Tcra-Tcrd locus in the combination of the Eα viewpoint (pale blue rectangle) and the SOI containing Trdd2 (pale red rectangle) in DN thymocytes from *Rag1* ^−/−^ and EACBE^−/−^ *Rag1* ^−/−^ mice.

To investigate the impact of EACBE on the higher-order chromatin structure of *Tcra*-*Tcrd* at the DN stage, we did a three-way interaction analysis recently developed in our laboratory. This method has previously been used to examine the higher-order chromatin architecture of the *Tcra*-*Tcrd* locus in DP thymocytes, as well as the cooperative interactions among Vα, Jα, and Eα (31). In this study, we applied this method to analyze the higher-order chromatin structure of the locus in DN cells. The *Trdd2* three-way contact heatmap showed that the deletion of EACBE resulted in a marked increase in interactions between INTs and TEAp with *Trdd2* in DN cells (**Figure 5A**). Furthermore, the KO – WT subtraction heatmap demonstrates a significant enhancement in three-way interactions between INTs and *Dad1* (**Figure 5B**), a pattern also observed in the Eα three-way contact heatmap (**Supplementary Figures S4A, S4B**). Examining the *Trdv2–2* three-way contact heatmap, we observed that co-occurring interaction pairs are confined in a small region surrounding *Trdv2–2* in WT DN cells and the EACBE deletion does not influence the three-way interaction from the viewpoint of *Trdv2-2* (**Supplementary Figure S4D**). However, the sequences interacting with the Eα-INT2 combination were significantly reduced, including the three-way contact involving Eα-INT2-Trdv2-2 (**Supplementary Figures S4A, S4B**). These results indicate that EACBE deletion increases the insulation of INTs.

To elucidate the relationship between EACBE and INTs in DN cells, we performed a method developed by Allahyar et al. to analyze specific three-way contacts. This method employs a second Site of Interest (SOI) to distinguish between preferred and random or disfavored three-way contacts (31, 33). First, we examined the co-occurrence frequency of third sequences across the *Tcra*-*Tcrd* locus when *Trdd2* interacts with INT2 as an SOI. Most sequences located between INTs and TEAp, such as E_δ_, are disfavored in three-way contacts with the *Trdd2*-INT2 combination (**Figure 5C**). However, some sequences upstream of INTs and surrounding TEAp are favored in three-way contacts with the *Trdd2*-INT2 combination (**Figure 5C**). Notably, EACBE deletion leads to a reduction in synergistic interactions of upstream sequences of INTs with the *Trdd2*-INT2 combination (**Figure 5C**). When *Trdd2* and *Trdv5* are used as the viewpoint-SOI combination, the coordinated behavior of sequences upstream of INTs diminishes, accompanied by a shift in sequences between INTs and TEAp (**Figure 5D**). Furthermore, the viewpoint-SOI analysis reveals that EACBE deletion also facilitates the synergistic interaction of the sequences from INTs to TEAp with the Eα-*Trdd2* or Eα-INT2 combinations (**Figure 5E**, **Supplementary Figure S4C**). These findings suggest that EACBE reduces the insulation of INTs, thereby facilitating the rearrangement of proximal V_δ_ segments.

## 4 Discussion

In this study, we investigated the role of the CTCF binding site EACBE in regulating *Tcrd* rearrangement and its subsequent effect on *Tcra* rearrangement. Our previous work demonstrated that EACBE, situated downstream of the *Tcra*-*Tcrd* locus, functions as a chromatin boundary that insulates the locus from the downstream region at the DP stage (28). We found that EACBE directly regulates *Tcrd* rearrangement during the DN stage. Specifically, EACBE facilitates the usage of proximal V_δ_ genes, such as *Trdv2–2* and *Trdv1*, while reducing the usage of *Trdv5* and distal V_δ_ genes. Additionally, the deletion of EACBE leads to increased rearrangement of Trav15 family members, which in turn enhances usage of central V_α_ genes during *Tcra* rearrangement (**Supplementary Figure S5**). These findings are consistent with a recent report by Danielle J et al., which found that the Trav15 family is a crucial contributor to *Tcra* repertoire diversity (27).

Notably, EACBE plays a crucial role in the diversity of Trav14-related TCRs. The Trav14 gene segments are located just upstream of the Trav15 family. Sleckman et al. reported that the V_α_ repertoires in peripheral T cells in E_α_-deficient mice were markedly restricted, characterized by a predominance of Trav14-related TCRs, in contrast to the 5%–10% of Trav14 usage observed in WT mice that express Trav14 family members (30). Based on our findings, this skewed usage can be attributed to the unchanged *Tcrd* rearrangement in E_α_-deficient DN cells. These cells frequently rearrange Trav15 segments but fail to differentiate into γδ T cells, instead progressing to DP cells. At the DP stage, rearranged Trav15 segments facilitate the spatial juxtaposition of Trav14 to J_α_, thereby promoting Trav14 usage in E_α_-deficient cells.

The effect of EACBE on *Tcra* rearrangement is complex. We previously reported that EACBE deletion affects the usage of J_α_, which is mainly caused by affecting the initiation of primary rearrangement (28). However, the effect of EACBE on V_α_ usage is more complex and can be affected directly and indirectly. The indirect effect comes from *Tcrd* rearrangement. EACBE deletion increases the usage of Trav15, so that the primary rearrangement of *Tcra* has more chances to start from the upstream of Trav15. In addition, the effect of EACBE deletion on *Tcra* primary rearrangement may also affect V_α_ usage, increase the usage of V_α_ genes proximal upstream of Trav15, and reduce the usage of V_α_ segments distal upstream of Trav15. Since Tcra can undergo multiple rounds of rearrangement, secondary rearrangement also plays an important role in shaping *Tcra* repertoire. It is generally believed that in secondary rearrangement, the linear distance between V_α_ and J_α_ segments are close, and their rearrangement is less affected by chromatin conformation. However, we cannot answer whether EACBE affects secondary rearrangement here. It needs to construct rearranged V_α_-J_α_ knockin on the EACBE deleted allele to answer this question.

EACBE also facilitates the rearrangement of proximal V_α_ by modulating interactions between E_α_ and proximal V_α_ segments in DP cells. In parallel, it can indirectly influence the rearrangement of central V_α_ segments by regulating *Tcrd* rearrangement at the DN stage. Nonetheless, it remains plausible that EACBE directly regulates the rearrangement of central Vα segments in alleles where the Tcrd gene is intact. Chen et al. previously showed that INTs function as insulators that segregate *Trdv2-2*, the most frequently used V_δ_ gene, from the D_δ_-J_δ_-C_δ_ region (26). This insulation promotes the usage of alternative V_δ_ segments, thereby contributing to the diversification of the *Tcrd* repertoire (34). These findings suggest that EACBE and INTs exert opposing influences on *Tcrd* rearrangement and indirectly shape the *Tcra* repertoire, thereby balancing the diversity of the *Tcra* and *Tcrd* repertoires.

Chromatin-organizing proteins such as cohesin and CTCF, along with their binding sites, are integral to the coordination of antigen receptor gene rearrangement. These proteins facilitate the generation of diverse antigen receptor repertoires by modulating the spatial conformation of chromatin (28, 41–43). Notably, no direct chromatin loop has been observed between *Trdv2–2* and *Trdd2*. However, our analysis of higher-order chromatin structures revealed that EACBE can attenuate the insulation ability of INTs, thereby enhancing interactions between the *Tcrd* recombination center and the upstream region, ultimately facilitating the rearrangement of proximal V_δ_ segments. This effect may be attributed to cohesin extrusion from EACBE towards the upstream region, promoting interactions across the INTs boundary and reducing its insulation.

In this study, we showed that EACBE deletion results in a modest reduction in chromatin activity at *Trdv2–2* and the *Tcrd* recombination center. Although E_α_ is not transcriptionally active during the DN stage, it is primed through the recruitment of constitutive transcription factors and the presence of the poised enhancer marker H3K4 mono-methylation (44–48). EACBE-mediated chromatin extrusion may facilitate the special proximity of E_α_ and its associated transcription factors to *Trdv2-2*, thereby enabling their engagement in the transcriptional regulation of *Trdv2–2* and promoting its rearrangement.

In conclusion, this study examined the role of EACBE on the *Tcrd* rearrangement and chromatin conformation of the *Tcra*-*Tcrd* locus at the DN stage. Our results indicate that EACBE diminishes the insulating ability of INTs, thereby promoting the rearrangement of proximal V_δ_ segments. Given the observation that INTs facilitate the rearrangement of distal V_δ_ segments, we conclude that EACBE and INTs collaboratively regulate the diversity of the *Tcrd* repertoire and subsequently indirectly influence the diversity of the *Tcra* repertoire. This research offers novel insights into the role of two distinct CTCF binding sites in the regulation of V(D)J recombination of the antigen receptor locus.

## Data Availability

The datasets presented in this study can be found in online repositories. The names of the repository/repositories and accession number(s) can be found in the article/**Supplementary Material**.
