# Supplementary material for: The insulator EACBE regulates V(D)J recombination of Tcrd gene by modulating chromatin organization
Source: Front Immunol. 2025 Jul 17;16:1613621. doi: 10.3389/fimmu.2025.1613621 (PMC12310595; doi:10.3389/fimmu.2025.1613621)
Supplement: Supplementary file 1 [file DataSheet1.docx]

Supplementary Material

**Title:** **The insulator EACBE regulates V(D)J recombination of Tcrd gene by modulating chromatin organization**

Yongchang Zhu^1, 2, #^, Ranran Dai^3, 4 #^, Hao Zhao^5^, Junwei Luo^5^, Keyi Li^5^, Wei Xue^5^, Litao Qin^1^, Hongyuan Pan^5^, Shixiu Liao^1^ *, Bingtao Hao^2, 6^ *

^1^Henan Key Provincial Laboratory of Genetic Diseases and Functional Genomics, People's Hospital of Zhengzhou University, Zhengzhou university, Zhengzhou, China

^2^Department of Immunology, School of Basic Medical Sciences, Zhengzhou University, Zhengzhou, China

^3^RNA Biomedical Institute, Sun Yat-Sen Memorial Hospital, Zhongshan School of Medicine, Sun Yat-Sen University, Guangzhou, China.

^4^Center for Stem Cell Biology and Tissue Engineering, Key Laboratory for Stem Cells and Tissue Engineering, Ministry of Education, Zhongshan School of Medicine, Sun Yat-Sen University, Guangzhou, China.

^5^Cancer Research Institute, School of Basic Medical Sciences, Southern Medical University, Guangzhou, China.

^6^Henan Eye Institute, Henan Academy of Innovations in Medical Science, Zhengzhou, China.

**^#^ same contribution**

*** Correspondence:**Bingtao Hao, Department of Immunology, School of Basic Medical, Zhengzhou University, No.100 Science Avenue, Zhengzhou City, Henan Province, China. E-mail: [haobt123@zzu.edu.cn](mailto:haobt123@163.com)

Shixiu Liao, Henan Provincial Key laboratory of Genetic Diseases and Functional Genomics, Henan Provincial People’s Hospital, Zhengzhou university, China. Email: ychslshx@zzu.edu.cn

# Supplementary Figures and Tables

## Supplementary Figures


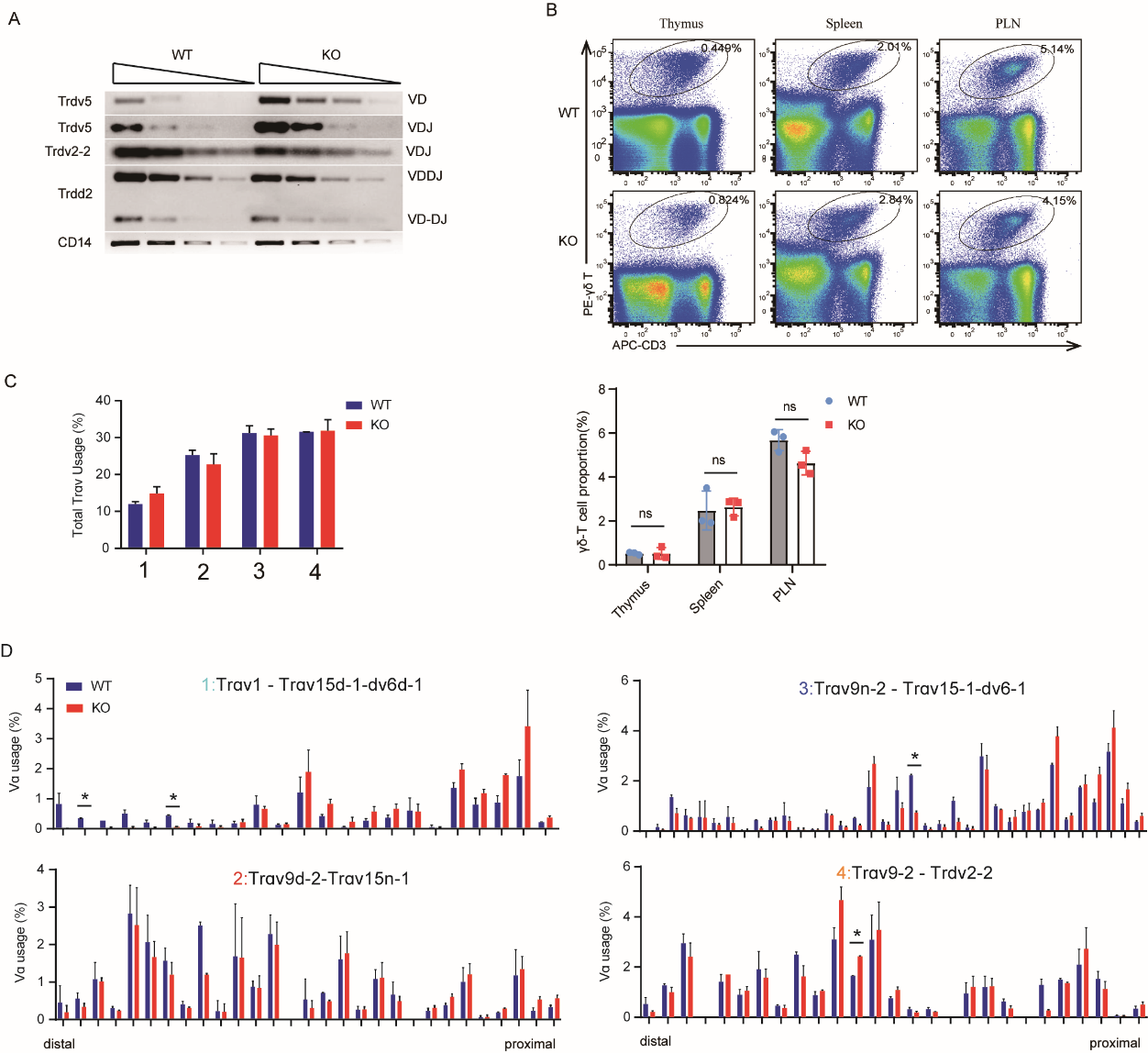


**Supplementary Figure 1. Related to Fig. 1 and Fig. 2**

**(A**) Tcrd rearrangement in two-fold serially diluted genomic DNA from wild type (WT) and EACBE^−/−^ thymocytes detected by PCR and visualized by Southern blotting with specific probes. Data are representative of two independent experiments.

**(B**) Flow cytometric analysis of the γδ T cell populations in the thymus, spleen, and lymph nodes of WT and EACBE^-/-^ mice. Data are representative of three independent experiments. ns P >0.05 by two side multiple Student’s *T* test.

**(C)** Total Vα usage of the four regions from Fig.1C, calculated from previous *Tcra* repertoire sequencing data (GEO: GSE145147).

**(D)** The usage frequency of each Vα in each region from Fig.1C, calculated from previous *Tcra* repertoire sequencing data (GEO: GSE145147). * P <0.05 by two side multiple Student’s *T* test.


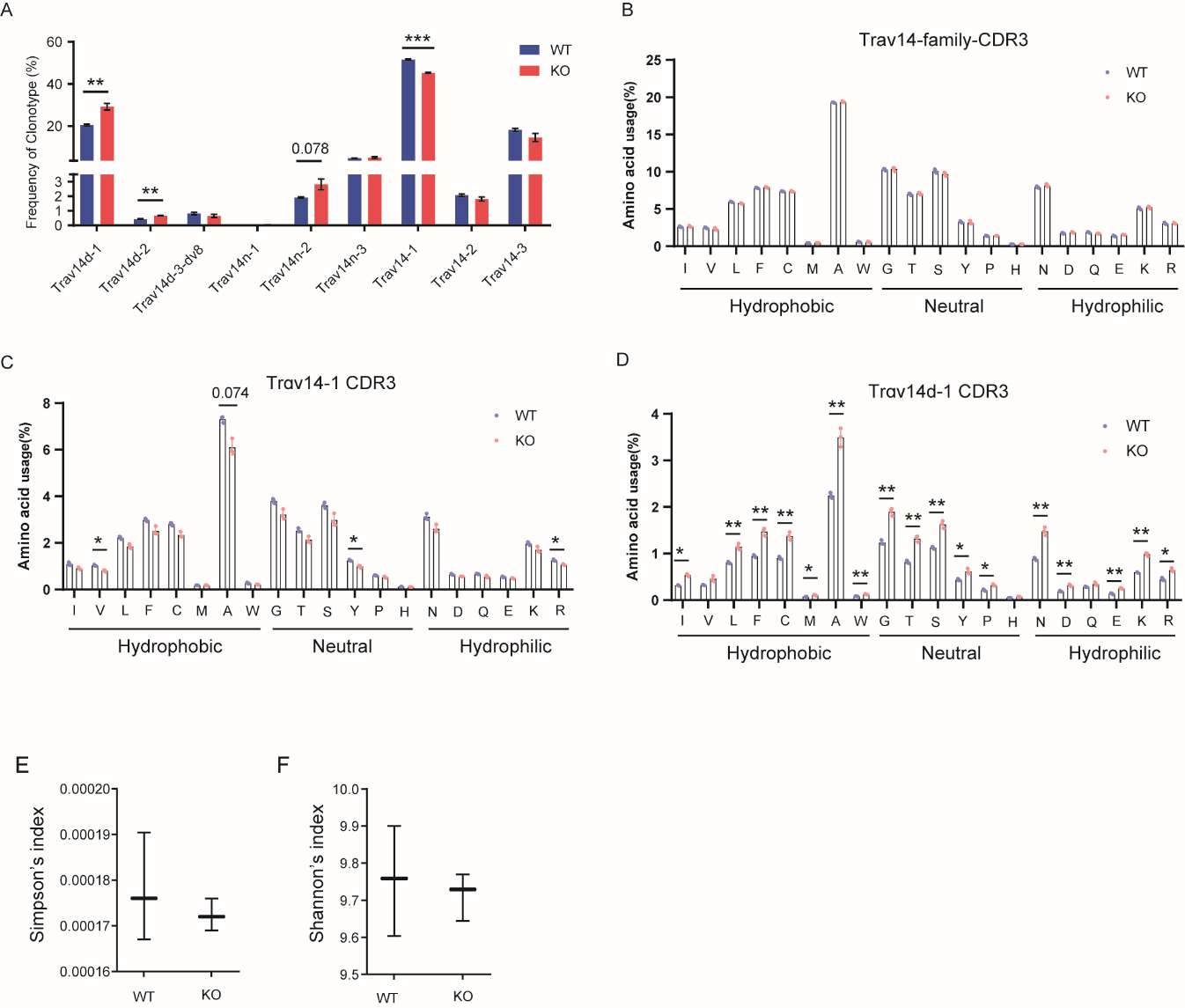


**Supplementary Figure 2. Related to Fig. 2**

**(A)** Histogram showing the clonotype frequency of each member of the Trav14 family from Fig.2A. Data represent the mean ± s.d. of three experiments. ** P <0.01, *** P <0.001 by two side multiple Student’s *T* test.

**(B)** The frequency of CDR3 amino acid usage of the Trav14 family-related TCRα chain from WT and EACBE^−/−^ sorted DP cells. Amino acids are ordered from the most hydrophilic one to the most hydrophobic according to the Kyte-Doolitlle scale. Data represent the mean ± s.d. of three experiments.

**(C-D)** The frequency of CDR3 amino acid usage of the *Trav14-1*(C) and *Trav14d-1*(D)-related TCRα chain from WT and EACBE^−/−^ sorted DP cells. Data represent the mean ± s.d. of three experiments. * P <0.05, ** P <0.01 by two side multiple Student’s *T* test.

**(E-F)** Simpson’s index (E) and Shannon’s index (F) of Trav14 family repertoires in WT and EACBE^-/-^ mice. Data represent the mean ± s.d. of three experiments.


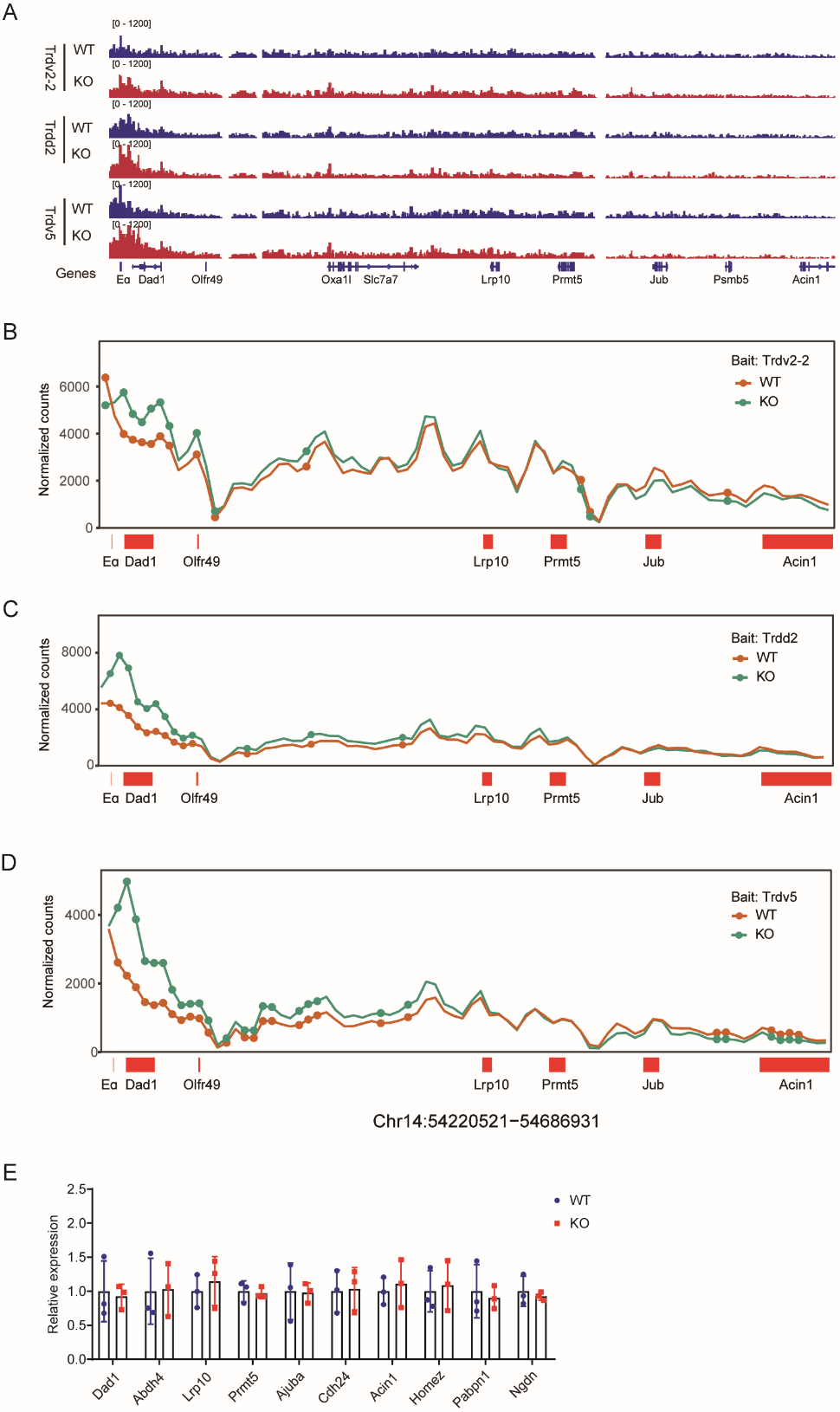


**Supplementary Figure 3.** **Related to Fig. 4**

**(A**) Genome browser views depicting 3C-HTGTS pairwise chromatin interactions from *Trdv2-2*, *Trdd2*, and *Trdv5* viewpoints in the downstream region of the *Tcra*-*Tcrd* locus in DN thymocytes from *Rag1*^−/−^ (WT, blue) and EACBE^−/−^ *Rag1*^−/−^ (KO, red) mice. 3C-HTGTS is representative of three replicates for each viewpoint. Gene annotations are shown below.

**(B-D)** Line plots displaying the difference of pairwise interactions between *Rag1*^−/−^ (WT, orange) and EACBE^−/−^ *Rag1*^−/−^(KO, green) mice at the *Trdv2-2* (B), *Trdd2* (C) and *Trdv5* (D) viewpoints using the 4C-ker program. Analysis is based on three independent experimental replicates. Filled circles highlight significant differential interactions (*P* < 0.05; statistics derived using DESeq2).

**(E)** Relative transcription of the genes in the downstream region of EACBE in DN thymocytes from *Rag1*^−/−^ and EACBE^−/−^ *Rag1*^−/−^ mice detected using reverse-transcription qPCR. Expressions were normalized to the *Actb* gene. Data represent the mean ± s.d. of three experiments.


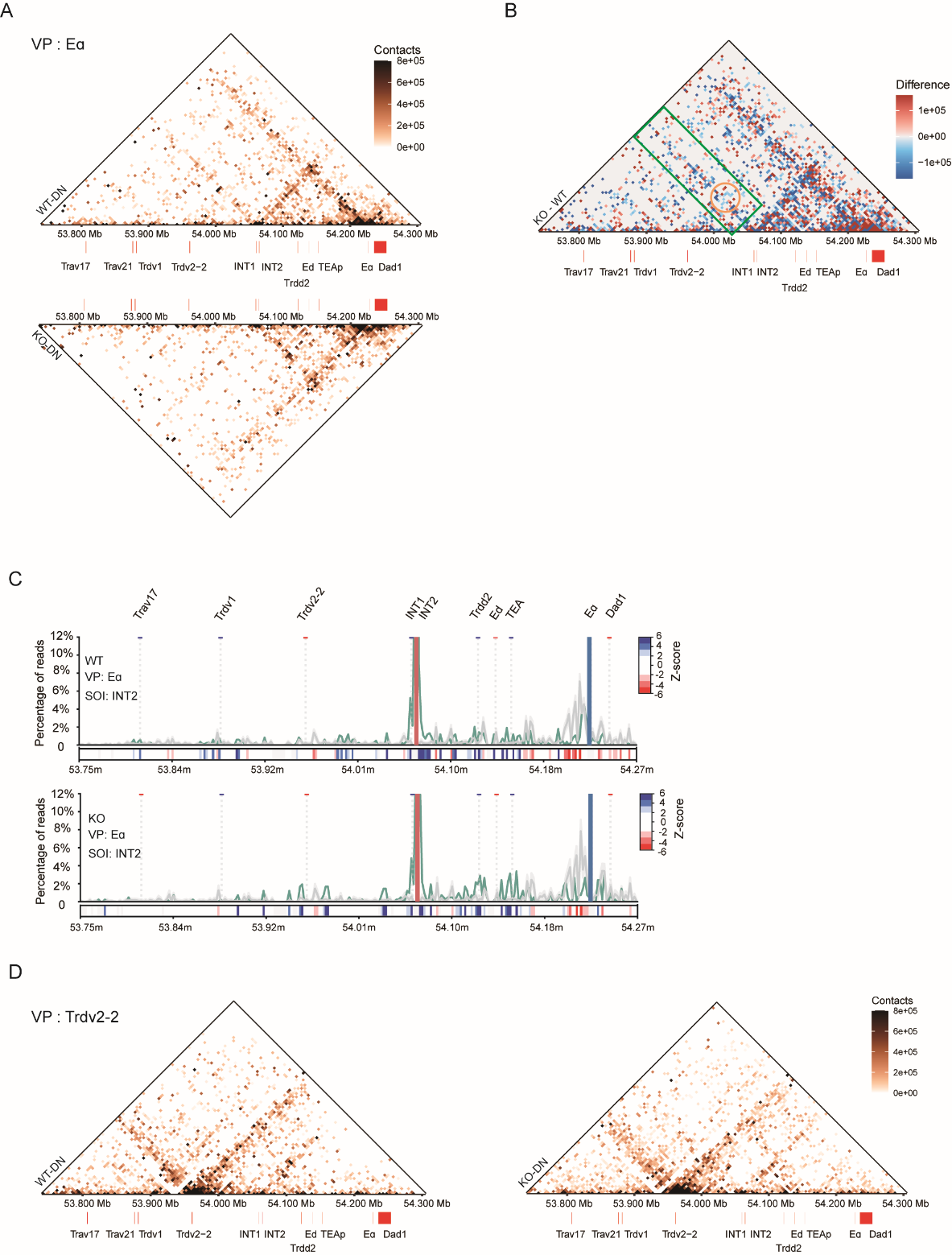


**Supplementary Figure 4.** **Related to Fig. 5**

**(A)** Heatmap showing three-way chromatin interactions in the 3’ portion of the Tcrα-Tcrd locus from the Eα viewpoint in DN thymocytes from *Rag1*^−/−^ (WT, up) and EACBE^−/−^ *Rag1*^−/−^ (KO, down) mice. The heatmap represents mean of three experimental replicates. Gene annotations are shown middle. Resolution: 5kb; Coordinates (mm10): chr14: 53738375-54282925.

**(B)** EACBE^-/-^ – WT subtraction heatmap (resolution: 5kb) showing the three-way contact differences from panel (A). Green rectangular displaying the sequences interacting with the Eα-INT2 combination. Orange circle representing the three-way contact involving Eα-INT2-Trdv2-2.

**(C)** VP-SOI plots displaying co-occurrence contacts of sequences in the 3’ portion of the Tcra-Tcrd locus in the combination of the Eα viewpoint (pale blue rectangle) and the SOI containing INT2 (pale red rectangle) in DN thymocytes from *Rag1*^−/−^ and EACBE^−/−^ *Rag1*^−/−^ mice. The green line represents the observed co-occurrence frequency, and the gray line represents the expected frequency (mean ± s.d.) of sequences across the locus. z-scores (dark blue) indicating significant.

**(D)** Heatmap showing three-way chromatin interactions in the 3’ portion of the Tcrα-Tcrd locus from the *Trdv2-2* viewpoint in DN thymocytes from *Rag1*^−/−^ (WT, left) and EACBE^−/−^ *Rag1*^−/−^ (KO, right) mice. The heatmap represents mean of three experimental replicates. Gene annotations are shown middle. Resolution: 5kb; Coordinates (mm10): chr14: 53738375-54282925.


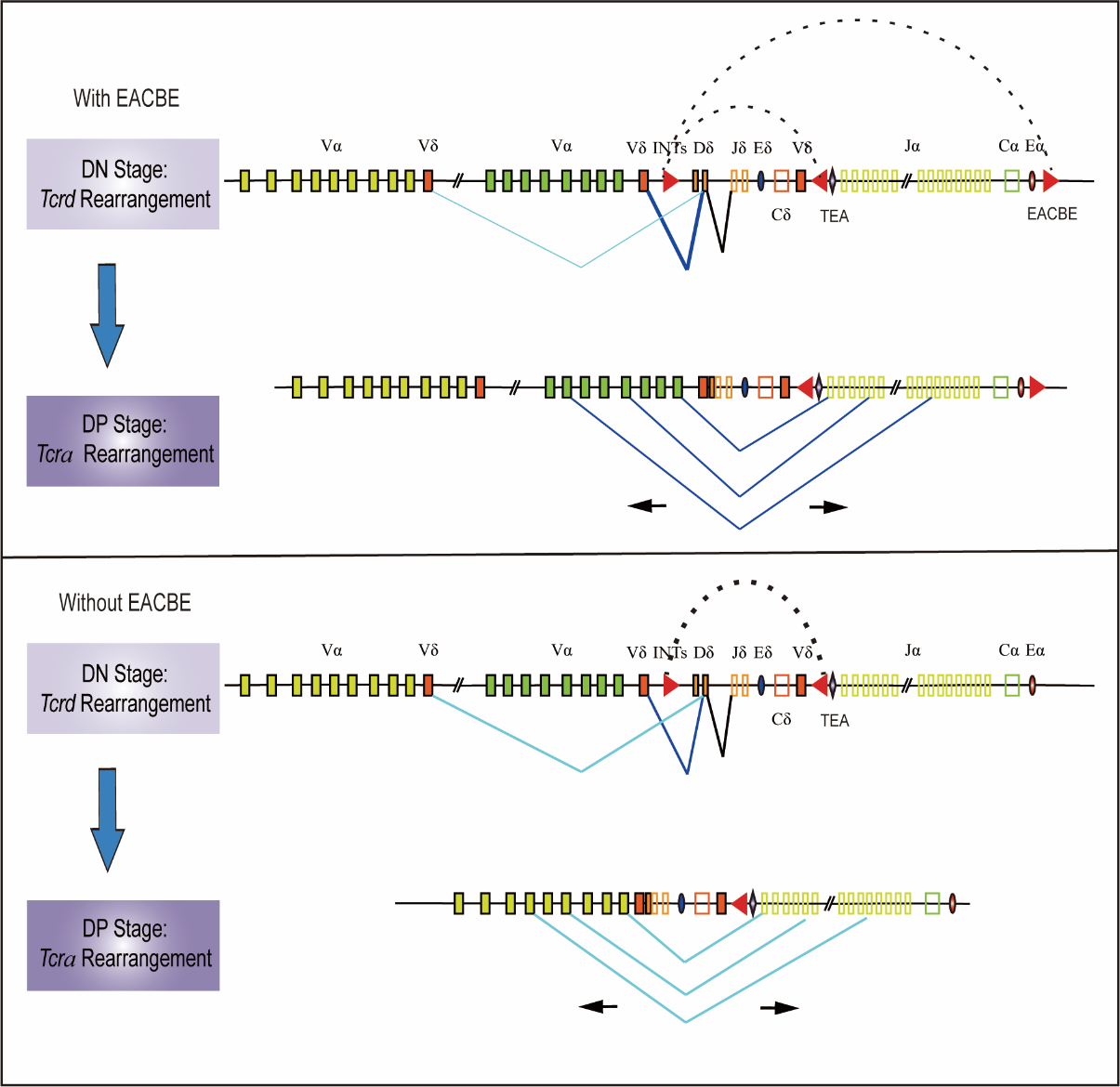


**Supplementary Figure 5.** **The schematic diagram illustrates that EACBE exerts an indirect influence on the rearrangement of *Tcra* by modulating the rearrangement of *Tcrd*.**

Red triangles signify the CTCF binding elements (CBEs), with their orientation reflecting the CBE direction. Dotted lines represent interactions between two loci, with their thickness indicating interaction strength. During the DN stage, the azure and navy-blue lines denote the rearrangement of Vδ and DJδ at various loci. In the DP stage, these lines illustrate the loci and progression of Vα initiation rearrangement after Vδ rearrangement. The line thickness indicates the rearrangement intensity. Black arrows depict the directionality of multiple rounds of *Tcra* gene rearrangement.

In brief, EACBE facilitates the rearrangement of proximal Vδ segments while concurrently restricting the rearrangement of distal Vδ segments in DN cells. The EACBE deletion results in an enhanced rearrangement of distal Vδ segments, which consequently leads to an increased usage of Vα upstream of these Vδ in DP cells.

## Supplementary Tables

**Table S1 Primer sequences for Vδ usage analysis**

| Primers | Sequence |
| --- | --- |
| DSB linker1 | GCGGTGACCCGGGAGATCTGAATTC |
| DSB linker2 | GAATTCAGATC |
| Llinker primer | CCGGGAGATCTGAATTCCAC |
| Cd14 F | GCTCAAACTTTCAGAATCTACCGAC |
| Cd14 R | AGTCAGTTCGTGGAGGCCGGAAATC |
| TRDV2-2 GF | CCGCTTCTCTGTGAACTTCC |
| TRDV5 GF | CAGATCCTTCCAGTTCATCC |
| TRDD2 GF | ATTCTCTGCCTAGCCCACACAG |
| TRDJ1 GR | CAGTCACTTGGGTTCCTTGTCC |

**Table S2 Primer sequences for 3C HTGTS assay**

| Bait | Nested Primer | Adapter Primer |
| --- | --- | --- |
| Eα | cttgttctgattggatggcga | GACTATAGGGCACGCGTGG |
| Trdd2 | tacaaagcccagggaaggtt | GACTATAGGGCACGCGTGG |
| Trdv2-2 | cttcctgtcattctgcacaga | GACTATAGGGCACGCGTGG |
| Trdv5 | gccaacagaaccttccatct | GACTATAGGGCACGCGTGG |

**Table S3 Primer sequences for HTGTS assay**

| Bait | Nested Primer | Adapter Primer |
| --- | --- | --- |
| Trdj1 | tggttccacagtcacttggg | GACTATAGGGCACGCGTGG |
| Traj61 | tgctgagtttcatgagtcttcc | GACTATAGGGCACGCGTGG |
| Trav14 | ACTCTCAGCCTGGAGACTCAG | GACTATAGGGCACGCGTGG |

**Table S4 Primer sequences for Germline transcription**

| Primers | Sequence |
| --- | --- |
| GT-Trav15-2/dv6-2-F | ctaaggatatcaaacagggagca |
| GT-Trav15-2/dv6-2-R | gaagcttatcctcttatagt |
| GT-Trav13-4/dv7-F | agcctcgtcagcctgttgtc |
| GT- Trav13-4/dv7-R | gaaatgtgcaaagagccgcgac |
| GT-Trav17-F | TGGAGCGACTCAGCCAAGTA |
| GT-Trav17-R | CGTGCACAGAAGGTCTCAGG |
| GT-Trdv2-2-F | tgaaggtgagacagtgctag |
| GT-Trdv2-2-R | agcaaatgggtttgccttctta |
| GT- Trdd1-F | TACGGCTGTGTTTCACTGTGAT |
| GT- Trdd1-R | GCTCAATGGACTCTTTGCAGTG |
| GT- Trdd2-F | acaaagcccagggaaggttt |
| GT- Trdd2-R | acggttcttcaccctgcagtt |
| GT- Trdj1-F | AGCTGCTGAGGTTTTTGGAATG |
| GT- Trdj1-R | ATCCCTCAGACCCTAACCCAGA |
| GT- Trdj2-F | GCTGGTCCCAGACTGGTTATCT |
| GT- Trdj2-R | AACTTACGGGGCTCCACAAAG |
| GT-Ed-F | tcaaccgcaaaatacatgcc |
| GT-Ed-R | agcattaacggctggaaacc |
| GT-Trdc-F | tggggaccagcttcattatc |
| GT-Trdc-R | ggctctggctagggatgagt |
| GT-Trdv5-F | AAGCTTCCGAAGGTCAGAGAGA |
| GT-Trdv5-R | ACCGACTGGAAGGATGATTCTT |
| GT-TEA-F | atcatccccgagactgatac |
| GT-TEA-R | cacaagatagctagtgtcctg |
| GT-Actb-F | ccctaggcaccaggtaagtg |
| GT-Actb-R | accatcacaccctgtggaag |

**Table S5 Primer sequences for transcription of the genes in the downstream region of EACBE**

| Primers | Sequence |
| --- | --- |
| Dad1 mRNA F | TGTGGGCAGCTTCATCCTAG |
| Dad1 mRNA R | GTGCTGGCAAAGAGGAAGTC |
| Abdh4 mRNA F | TGGAAGCCAGGATCCTCCAG |
| Abdh4 mRNA R | CATCACCAGAGGGGTGCGAT |
| Ajuba mRNA F | TGCTCTGCCCATAGATACCT |
| Ajuba mRNA R | GTCTCCTGGTCCCTTCGTTC |
| Cdh24 mRNA R | CGTCTTCGGGCTCAATGGA |
| Cdh24 mRNA F | GGGCCAGATCTCAGCCAGT |
| Prmt5 mRNA R | GGTGGTTGGTTCCCGTGATG |
| Prmt5 mRNA F | GCCATTCTCCCCACCAGCAT |
| Acin1 mRNA F | GATGGAGCTGCAGCCTCCT |
| Acin1 mRNA R | CCCGTTCCGCGTCAAGCAG |
| Lrp10 mRNA F | GACCGGATCACTTTCCCACG |
| Lrp10 mRNA R | TGCAGTTGGCAGGGGAGCT |
| Homez mRNA F | AGCAGGTGCTCATTTCCATCC |
| Homez mRNA R | AGCAGTCTCAACAGCTCTGCA |
| Pabpn1 mRNA F | TCAAAGCTCGAGTCAGGGAGA |
| Pabpn1 mRNA R | ACGTAGATAGAGCGGGCATCA |
| Ngdn mRNA F | CACTGACGACAAAAGTTCGAGC |
| Ngdn mRNA R | AGAGGCCTTGTCCAGGATGA |

**Table S6 Primer sequences for ChIP-qPCR**

| Primer | Sequence |
| --- | --- |
| Trav21-F | TGTGGGGTTGCTGCTTGAAG |
| Trav21-R | AACACTTACCCAAAGCCAGGAG |
| Trdv2-2-F | TCCTGTTTTGAAGGTGAGACAG |
| Trdv2-2-R | GCCTTCTTACCAAGAGAAGTGG |
| Trdd1-F | TACGGCTGTGTTTCACTGTGAT |
| Trdd1-R | GCTCAATGGACTCTTTGCAGTG |
| Trdj1-F | AGCTGCTGAGGTTTTTGGAATG |
| Trdj1-R | ATCCCTCAGACCCTAACCCAGA |
| Trdj2-F | GCTGGTCCCAGACTGGTTATCT |
| Trdj2-R | AACTTACGGGGCTCCACAAAG |
| Trdv5-F | CTGGACTCTCTTTAACCATCCC |
| Trdv5-R | TCCTTGGTAGAGGTATTCACCC |
| TEAp-F | ATGGGAAAGGGACCGATAAG |
| TEAp-R | GCTCAAAGGACACTGGAAGG |
| Ea-F | TTCCAGCGGGATACCTGTTA |
| Ea-R | ACCCCTTTGGCCATTTCTTA |
| Actb-F | ACGATGGAGGGGAATACAGC |
| Actb-R | TGATAGTTCGCCATGGATGAC |

**Table S7 Deposited data**

| Data | Source | Accession |
| --- | --- | --- |
| EACBEwt-Rag1ko-DN-Ea-3C-HTGTS-rep1 | This paper | GEO: GSE265888 |
| EACBEwt-Rag1ko-DN-Ea-3C-HTGTS-rep2 | This paper | GEO: GSE265888 |
| EACBEwt-Rag1ko-DN-Ea-3C-HTGTS-rep3 | This paper | GEO: GSE265888 |
| EACBEko-Rag1ko-DN-Ea-3C-HTGTS-rep1 | This paper | GEO: GSE265888 |
| EACBEko-Rag1ko-DN-Ea-3C-HTGTS-rep2 | This paper | GEO: GSE265888 |
| EACBEko-Rag1ko-DN-Ea-3C-HTGTS-rep3 | This paper | GEO: GSE265888 |
| EACBEwt-Rag1ko-DN-Trdv5-3C-HTGTS-rep1 | This paper | GEO: GSE265888 |
| EACBEwt-Rag1ko-DN-Trdv5-3C-HTGTS-rep2 | This paper | GEO: GSE265888 |
| EACBEwt-Rag1ko-DN-Trdv5-3C-HTGTS-rep3 | This paper | GEO: GSE265888 |
| EACBEko-Rag1ko-DN-Trdv5-3C-HTGTS-rep1 | This paper | GEO: GSE265888 |
| EACBEko-Rag1ko-DN-Trdv5-3C-HTGTS-rep2 | This paper | GEO: GSE265888 |
| EACBEko-Rag1ko-DN-Trdv5-3C-HTGTS-rep3 | This paper | GEO: GSE265888 |
| EACBEwt-Rag1ko-DN-Trdd2-3C-HTGTS-rep1 | This paper | GEO: GSE265888 |
| EACBEwt-Rag1ko-DN-Trdd2-3C-HTGTS-rep2 | This paper | GEO: GSE265888 |
| EACBEwt-Rag1ko-DN-Trdd2-3C-HTGTS-rep3 | This paper | GEO: GSE265888 |
| EACBEko-Rag1ko-DN-Trdd2-3C-HTGTS-rep1 | This paper | GEO: GSE265888 |
| EACBEko-Rag1ko-DN-Trdd2-3C-HTGTS-rep2 | This paper | GEO: GSE265888 |
| EACBEko-Rag1ko-DN-Trdd2-3C-HTGTS-rep3 | This paper | GEO: GSE265888 |
| EACBEwt-Rag1ko-DN-Trdv2-2-3C-HTGTS-rep1 | This paper | GEO: GSE265888 |
| EACBEwt-Rag1ko-DN-Trdv2-2-3C-HTGTS-rep2 | This paper | GEO: GSE265888 |
| EACBEwt-Rag1ko-DN-Trdv2-2-3C-HTGTS-rep3 | This paper | GEO: GSE265888 |
| EACBEko-Rag1ko-DN-Trdv2-2-3C-HTGTS-rep1 | This paper | GEO: GSE265888 |
| EACBEko-Rag1ko-DN-Trdv2-2-3C-HTGTS-rep2 | This paper | GEO: GSE265888 |
| EACBEko-Rag1ko-DN-Trdv2-2-3C-HTGTS-rep3 | This paper | GEO: GSE265888 |
| EACBEwt-Rag1ko-DN-ATAC-rep1 | This paper | GEO: GSE265888 |
| EACBEwt-Rag1ko-DN-ATAC-rep2 | This paper | GEO: GSE265888 |
| EACBEwt-Rag1ko-DN-ATAC-rep3 | This paper | GEO: GSE265888 |
| EACBEko-Rag1ko-DN-ATAC-rep1 | This paper | GEO: GSE265888 |
| EACBEko-Rag1ko-DN-ATAC-rep2 | This paper | GEO: GSE265888 |
| EACBEwt-DN-Trdj1-HTGTS-rep1 | This paper | GEO: GSE265888 |
| EACBEwt-DN-Trdj1-HTGTS-rep2 | This paper | GEO: GSE265888 |
| EACBEwt-DN-Trdj1-HTGTS-rep3 | This paper | GEO: GSE265888 |
| EACBEwt-DN-Trdj1-HTGTS-rep44 | This paper | GEO: GSE265888 |
| EACBEko-DN-Trdj1-HTGTS-rep1 | This paper | GEO: GSE265888 |
| EACBEko-DN-Trdj1-HTGTS-rep2 | This paper | GEO: GSE265888 |
| EACBEko-DN-Trdj1-HTGTS-rep3 | This paper | GEO: GSE265888 |
| EACBEko-DN-Trdj1-HTGTS-rep4 | This paper | GEO: GSE265888 |
| EACBEwt-DP-Traj61-HTGTS-rep1 | This paper | GEO: GSE265888 |
| EACBEwt-DP-Traj61-HTGTS-rep2 | This paper | GEO: GSE265888 |
| EACBEwt-DP-Traj61-HTGTS-rep3 | This paper | GEO: GSE265888 |
| EACBEko-DP-Traj61-HTGTS-rep1 | This paper | GEO: GSE265888 |
| EACBEko-DP-Traj61-HTGTS-rep2 | This paper | GEO: GSE265888 |
| EACBEko-DP-Traj61-HTGTS-rep3 | This paper | GEO: GSE265888 |
| EACBEwt-DP-Trav14-HTGTS-rep1 | This paper | GEO: GSE265888 |
| EACBEwt-DP-Trav14-HTGTS-rep2 | This paper | GEO: GSE265888 |
| EACBEwt-DP-Trav14-HTGTS-rep3 | This paper | GEO: GSE265888 |
| EACBEko-DP-Trav14-HTGTS-rep1 | This paper | GEO: GSE265888 |
| EACBEko-DP-Trav14-HTGTS-rep2 | This paper | GEO: GSE265888 |
| EACBEko-DP-Trav14-HTGTS-rep3 | This paper | GEO: GSE265888 |
| EACBEwt-Rag1ko-DN-GT-RNASeq-rep2 | This paper | GEO: GSE265888 |
| EACBEko-Rag1ko-DN-GT-RNASeq-rep1 | This paper | GEO: GSE265888 |
| CTCF ChIP-seq (DN) | ([Torkamani A](https://www.ncbi.nlm.nih.gov/pubmed/?term=Torkamani%20A%5bAuthor%5d), et al., 2012) | GEO: GSM 1023416 |
| RACE-Rep1-WT-TCRA | (Hao Zhao et al., 2020) | GEO: [GSM4307229](https://www.ncbi.nlm.nih.gov/geo/query/acc.cgi?acc=GSM4307209) |
| RACE-Rep2-WT-TCRA | (Hao Zhao et al., 2020) | GEO: [GSM4307230](https://www.ncbi.nlm.nih.gov/geo/query/acc.cgi?acc=GSM4307210) |
| RACE-Rep1-KO-TCRA | (Hao Zhao et al., 2020) | GEO: [GSM4307223](https://www.ncbi.nlm.nih.gov/geo/query/acc.cgi?acc=GSM4307209) |
| RACE-Rep2-KO-TCRA | (Hao Zhao et al., 2020) | GEO: [GSM4307224](https://www.ncbi.nlm.nih.gov/geo/query/acc.cgi?acc=GSM4307210) |
